# Anticancer Effects of Rosemary (*Rosmarinus officinalis* L.) Extract and Rosemary Extract Polyphenols

**DOI:** 10.3390/nu8110731

**Published:** 2016-11-17

**Authors:** Jessy Moore, Michael Yousef, Evangelia Tsiani

**Affiliations:** 1 Department of Health Sciences, Brock University, St. Catharines, ON L2S 3A1, Canada; jessy.moore@brocku.ca (J.M.); my11dq@brocku.ca (M.Y.); 2 Centre for Bone and Muscle Health, Brock University, St. Catharines, ON L2S 3A1, Canada

**Keywords:** rosemary extract, carnosic acid, rosmarinic acid, cancer, proliferation, survival, cell signaling

## Abstract

Cancer cells display enhanced growth rates and a resistance to apoptosis. The ability of cancer cells to evade homeostasis and proliferate uncontrollably while avoiding programmed cell death/apoptosis is acquired through mutations to key signaling molecules, which regulate pathways involved in cell proliferation and survival. Compounds of plant origin, including food components, have attracted scientific attention for use as agents for cancer prevention and treatment. The exploration into natural products offers great opportunity to evaluate new anticancer agents as well as understand novel and potentially relevant mechanisms of action. Rosemary extract has been reported to have antioxidant, anti-inflammatory, antidiabetic and anticancer properties. Rosemary extract contains many polyphenols with carnosic acid and rosmarinic acid found in highest concentrations. The present review summarizes the existing in vitro and in vivo studies focusing on the anticancer effects of rosemary extract and the rosemary extract polyphenols carnosic acid and rosmarinic acid, and their effects on key signaling molecules.

## 1. Introduction

Arguably the most fundamental traits of cancer cells are their enhanced proliferative and decreased apoptotic capacities [1]. Normal cells tightly control the production and release of growth factors, which regulate cell growth/proliferation, thereby ensuring cellular homeostasis and maintenance of normal tissue architecture. In cancer cells, these signals are deregulated and thus, homeostasis within the cell is disrupted. Proliferation of cancer cells may be enhanced in a number of ways. Cancer cells may produce growth factors to which they can respond via the expression of cognate receptors. The level of receptor proteins displayed on the surface of cancer cells can also be elevated, rendering these cells hyperresponsive to growth factors; the same outcome can result from alterations to the receptor molecules that facilitate activation of downstream signaling pathways independent of growth factor binding [1]. Alternatively, cancer cells can signal normal neighbouring cells resulting in mutations/alterations in signaling pathways. These alterations stimulate the release of growth factors which are supplied back to the cancer cells, enhancing their proliferation [2,3]. Growth factor receptors (GFR), such as epidermal GFR (EGFR) are plasma membrane proteins with intrinsic tyrosine kinase (TK) activity. Growth factor binding enhances the tyrosine kinase activity of the receptor causing receptor autophosphorylation. The phosphorylated tyrosine residues of the receptor act as docking sites for intracellular proteins containing Src-homology 2 (SH2) domains, leading to stimulation of intracellular signaling cascades such as the phosphatidylinositol 3-kinase (PI3K-Akt) and the Ras-mitogen activated protein kinase (Ras-MAPK) cascades, that result in enhanced proliferation and inhibition of apoptosis/enhanced survival.

The development of cancer is divided into three stages: initiation, promotion and progression. Initiation involves a change to the genetic makeup of a cell which primes the cell to become cancerous. During the stage of promotion various factors permit a single mutated cell to survive (resist apoptosis) and replicate, promoting growth of a tumor. Finally, as the cancerous cell replicates and develops into a tumor, the disease state progresses. As normal, healthy cells progress to a neoplastic state they acquire a series of hallmark capabilities which enable them to become malignant. The 6 hallmarks of cancer proposed by Hanahan and Weinberg include sustaining proliferative signaling, evading growth suppressors, resisting cell death, enabling replicative immortality, inducing angiogenesis, and activating invasion and metastasis [1]. As tumors progress and become more aggressive they will begin to exhibit more of these hallmarks. Current anticancer agents may be classified as chemopreventive or chemotherapeutic depending on which stage of carcinogenesis they target. To explore the chemopreventive potential of anticancer agents, cells in culture or animal models can be exposed to an anticancer agent before being exposed to a carcinogen. This provides evidence of the effect of an anticancer agent on the initiation and promotion stages of cancer. Alternatively, cells in culture or animal models may be exposed to a carcinogen to establish a neoplastic state prior to being treated with an anticancer agent and this provides evidence of the effect of an anticancer agent on the progression of cancer.

Many pharmaceutical agents have been discovered by screening natural products from plants. Some of these drugs such as the chemotherapeutics etoposide, isolated from the mandrake plant and Queen Anne’s lace, and paclitaxel and docetaxel, isolated from the wood and bark of the Nyssaceae tree, are currently successfully employed in cancer treatment [4]. The exploration into natural products offers great opportunity to evaluate new chemical classes of anticancer agents as well as study novel and potentially relevant mechanisms of action. Many labs, including ours have shown metformin, a drug derived from the lilac, has anticancer properties [5]. In addition, the polyphenol resveratrol, found in high concentrations in wine, has been shown to have anticancer effects in vitro and in vivo [6,7,8,9,10]. Importantly, metformin and resveratrol exhibit both chemopreventive and chemotherapeutic effects.

The plant *Rosmarinus Officinalis* L. a member of the mint family *Lamiaceae*, is native to the Mediterranean region and has many culinary and medicinal uses. The main polyphenols found in rosemary extract (RE) include the diterpenes carnosic acid (CA) and rosmarinic acid (RA) [11]. Rosemary extract and its polyphenols CA and RA have recently been explored and found to exert potent anticancer effects (reviewed recently in [12,13,14]). To establish a systematic literature review we used the online search engine Pubmed. We searched the key phrases: rosemary extract and cancer, carnosic acid and cancer, rosmarinic acid and cancer. We also included subtypes of cancer such as breast cancer, colon cancer, etc., as keywords in our search. All studies pertaining to our topic and published after the year 2000 were included in the current review. In the following sections, in vitro and in vivo studies on the effects of RE and its main polyphenols have been summarized and sorted by cancer cell type, in chronological order from earliest to most recent. Chronology was chosen as the sorting method to highlight how the literature has progressed and what knowledge is currently available. Initially we focused on the studies examining the anticancer effects of RE, we then highlighted studies in which mechanisms of action have been investigated and separately summarized the studies using the polyphenols CA and RA. The studies presented in the text are also summarized, organized and presented in a table format to allow the reader to extract the information easily.

This is a comprehensive systematic review and adds to the existing literature by summarizing all relevant studies using RE, CA and RA in each cancer subtype. The review is organized by experimental treatment (RE, CA, RA), type of cancer (histology) and the study model (in vitro or in vivo) resulting in a clear, detailed and inclusive summary of the existing literature. This review also focuses on the mechanistic data provided by these studies, which will be beneficial for future research to help focus efforts on identifying the main mechanisms involved in the anticancer action of RE, CA and RA.

## 2. Anticancer Effects of Rosemary Extract (RE): In Vitro Studies

Several in vitro studies using colon cancer cell lines have shown RE to exhibit anticancer properties (Table 1). Exposure of CaCo-2 colon cancer cells to RE drastically decreased colony formation at 30 µg/mL (24 h) [15]. Yi, et al. (2011) examined the anti-tumorigenic effect of several culinary and medicinal herbs on SW480 colon cancer cells and found RE to significantly decrease cell growth at a concentration of 31.25 µg/mL (48 h), with an IC50 of approximately 71.8 µg/mL [16]. Cell proliferation was dramatically decreased and cell cycle arrest was induced in HT-29 and SW480 cells using extracts that were standardized to CA (25%–43%) or to total polyphenol content (10 µM) [17,18,19]. Cell growth of SW620 and DLD-1 colon cancer cells was significantly inhibited by RE at 30 µg/mL (48 h), with an IC50 as low as 34.6 µg/mL. Furthermore, RE enhanced the inhibitory effects of the chemotherapeutic drug 5-fluorouracil (5-FU) on proliferation and sensitized 5-FU resistant cells [20].

In SW620 and DLD-1 colon cancer cells RE inhibited cell viability dose-dependently resulting in significant inhibition at concentrations as low as 20 µg/mL, and an IC50 around 25 µg/mL (48 h). This study used 5 different RE’s, containing increasing levels of carnosol (CN: 1%–3.8% *w*/*w*) and CA (10%–30% *w*/*w*). Inhibition of cell viability was correlated with increasing CA content. Furthermore, CA alone (at doses found in RE) decreased cell viability and this effect was potentiated by the addition of CN (at doses found in RE). However, the inhibition seen using RE was greater than the response seen with CA or CN alone or in combination suggesting that chemicals other than CA and CN present in RE, also contribute to its anticancer effects [21]. Similarly, RE inhibited cell viability in HT29, SW480 and HGUE-C-1 colon cells at comparable doses (1.5–100 µg/mL; 48 h) and the authors reported that individual fractions of RE containing CA and other polyphenols, while potent, were not as potent as the complete extract [22]. Using HCT116 and SW480 cells, 10–100 µg/mL RE standardized to 23% CA (24–72 h) inhibited cell viability and induced apoptosis [23]. Valdes, et al. have shown, using HT-29 colon cells, that 30–60 µg/mL RE (24–72 h) inhibits cell proliferation (IC50 16.2 µg/mL). Moreover, RE induced cell cycle arrest, necrosis, cholesterol accumulation and ROS accumulation [24,25,26]. These studies provide evidence for the role of RE as an anticancer agent in colon cancer cells, capable of consistently inhibiting cell growth and viability at relatively low concentrations in the 20–100 µg/mL range.

In rat RINm5F insulinoma cells, RE significantly inhibited cell proliferation at 25 µg/mL (24 h), viability at 12 µg/mL (24 h) and increased apoptosis at 25 µg/mL (24 h) [27] (Table 2). Exposure of pancreatic cancer cells PANC-1 and MIA-PaCa-2 to RE containing increasing concentrations of CN (1%–3.8% *w*/*w*) and CA (10%–30% *w*/*w*) resulted in significant inhibition of cell viability with an IC50 of 50 µg/mL (48 h) and 30 µg/mL (48 h) respectively. The RE containing 25.66% *w*/*w* CA (sub-max) caused maximal inhibition compared to other RE’s in PANC-1 cells, significantly inhibiting cell viability to approximately 60% at 40 µg/mL (48 h) [21].

Breast cancer can be classified under three subtypes based on the sensitivity of the tumors to chemotherapeutic agents. The subtypes are (i) estrogen receptor positive (ER+), which express ERα and therefore respond to estrogens; (ii) human epidermal growth factor receptor 2 positive (HER2+) which overexpress HER2 and can be either ER+ or ER−; (iii) triple negative (TN) which lack expression of ERα, progesterone receptor and HER2. One study used MCF-7 (ER+) breast cancer cells and a cigarette smoke solution (in PBS) collected from a cigarette with or without 40 mg RE added to the filter. The control used in this experiment was cells stimulated with 2.5 µM benzopyrene for 12–18 h and exposed to 1:19 *v*/*v* cigarette smoke solution for 2 h without an RE filter. The presence of RE in the filter lead to considerably reduced benzopyrene levels and associated DNA adduct formation [28] (Table 2).

RE inhibited cell proliferation in breast cancer cells with an IC50 of 90 µg/mL and 26.8 µg/mL in MCF-7 (ER+) and MDA-MB-468 (TN) cell lines respectively [29] (Table 2). In a similar study, dose-dependent inhibition of cell viability by 6.25–50 µg/mL (48 h) RE was seen in MDA-MB-231 (TN) and MCF-7 (ER+) breast cancer cells and MCF-7 cells had an IC50 of ~24.02 µg/mL. There is a discrepancy seen in the reported IC50 values which may be attributed to the different extraction methods used for the preparation of rosemary extract; supercritical CO_2_ [30] and ethanol extraction [29]. Furthermore, MCF-7 cells were used in 2 additional studies and while both were found to inhibit cell proliferation, the IC50 values varied greatly from 187 µg/mL [31] to 9.95–13.89 µg/mL (RE standardized to 25%–43% CA) [18]. In agreement with the aforementioned studies, the RE resulting in a higher IC50 value was obtained from an alcohol based, methanol extraction [31].

The effects of RE at 1–120 µg/mL (48 h) were explored in all three breast cancer subtypes, ER+, HER2+ and TN. RE caused dose-dependent inhibition of cell viability in all subtypes of breast cancer cells. Furthermore RE enhanced the effectiveness of the monoclonal antibody (mAb) trastusumab and the chemotherapeutic drugs tamoxifen and paclitaxel, used in the treatment of breast cancer [32]. Taken together, these studies suggest a role for RE to inhibit pancreatic and breast cancer cell viability and proliferation, and induce apoptosis at concentrations in the 10–100 µg/mL range.

Rosemary extract (6.25–50 µg/mL; 48 h) inhibited viability of DU145 and PC3 prostate cancer cells [30] (Table 3). In agreement with these data, significant inhibition of LNCaP and 22RV1 prostate cancer cell proliferation and viability, and an induction of apoptosis were seen with RE (50 µg/mL standardized to 40% CA; 24–48 h) [33]. RE was able to combat the enhanced prostate specific antigen (PSA) levels measured in cell culture media, indicative of prostate cancer, inhibiting levels to less than a fifth of what was seen in the control group. Correspondingly, levels of the androgen receptor, to which PSA binds, were significantly decreased by 50 µg/mL RE [33]. The inhibitory effects on both androgen sensitive and insensitive cell lines are important and suggest potential chemotherapeutic effects in different prostate cancer subtypes.

Using 5637 bladder cancer cells Mothana, et al. (2011) showed that RE inhibited cell proliferation with an IC50 of 48.3 µg/mL (48 h) [31] (Table 3). Exposure of A2780 ovarian cancer cells to 0.08% (0.8 mg/mL; 48 h) RE containing media resulted in significant inhibition of proliferation and induction of apoptosis and cell cycle arrest. Cisplatin is a chemotherapeutic agent used often in cancer treatment however, as with many chemotherapeutics, patients often develop resistance to treatment. At 0.08% RE enhanced the sensitivity of A2780 and cisplatin-resistant A2780CP70 cell lines to growth inhibition by cisplatin treatment, suggesting that RE may be of use in combination with cisplatin or potentially other chemotherapeutic drugs in patients who have developed an acquired resistance [34]. In HeLa cervical cancer cells, RE inhibited cell proliferation with an IC50 of 23.31 µg/mL (72 h) [35] and RE standardized to CA (25%–43%) inhibited cell proliferation with an IC50 of ~10 µg/mL (48 h) [18], suggesting that standardized extracts containing higher concentrations of CA may have greater anticancer effects. Furthermore, in human ovarian cancer cells SK-OV3 and HO-8910 rosemary essential oil (0.0625%–1%) inhibited cell viability with an IC50 of 0.025% and 0.076% in each cell line respectively (48 h) (Table 3) [36]. This study noted that the rosemary essential oil was more potent than its individual components (α-pinene, β-pinene, 1,8-cineole) when tested alone at the same concentrations.

In human liver Hep-3B cells, RE at 0–50 µg/mL (24–48 h) dose-dependently decreased cell viability [30,37] with an IC50 of 22.88 µg/mL [30] (Table 4). Cell viability was inhibited in Bel-7402 liver cells by rosemary essential oil with an IC50 of 0.13% (1.3 mg/mL; 48 h) [36] and in HepG2 liver cells by RE with an IC50 of 42 µg/mL (48 h) [38]. The latter study also found that of the 4 different extracts tested, those with higher concentrations of CA resulted in more potent inhibition of cell proliferation [38]. In lung cancer cells, RE decreased viability of NCI-H82 small cell carcinoma cells (6.25–50 µg/mL; 48 h) [30] and decreased proliferation of A549 non-small cell carcinoma cells (2.5–200 µg/mL) [39] with an IC50 or 24.08 µg/mL and 15.9 µg/mL in each cell line respectively (Table 4). In a V79 normal hamster lung fibroblast cell line RE was cytotoxic at 30 µg/mL (24 h) [15]. The cytotoxicity of RE in normal fibroblasts raises questions about its potential as a successful treatment option however, further research is required to fully examine the cytotoxicity issue in normal tissues.

Vitamin D analogues (VDA) are commonly used in clinical differentiation therapy of acute myeloid leukemia (AML) to attempt to restore a defect in the capacity of myeloid progenitor cells to mature into non-replicating adult cells. However, pharmacologically relevant doses have been found to result in many adverse events such as hypercalcemia and attempts to circumvent these adverse events have been unsuccessful. RE containing 10 µM equivalent of CA, or 10 µM CA alone (96 h) potentiated the ability of vitamin D derivatives to inhibit cell viability and proliferation, induce apoptosis and cell cycle arrest and increase differentiation of WEHI-3BD murine leukemic and human HL-60 leukemic cells [40,41] (Table 5). A study examining the human leukemia HL-60 and K-562 cell lines and the murine RAW264.7 macrophage/monocyte cell line found significant inhibition of proliferation with an IC50 of 0.14% (1.4 mg/mL) and 0.25% (2.5 mg/mL) for the HL-60 and K-562 cells, respectively. In addition 0.1% (1 mg/mL; 72 h) RE significantly increased differentiation of HL-60 cells [29]. RE inhibited viability at 50 µg/mL (48 h) in K-562 leukemia cells [30]. Similar effects of RE (50 µg/mL; 24 h) were reported by others that lead to decreased proliferation of K-562 cells [42].

## 3. Anticancer Effects of Rosemary Extract (RE): In Vivo Animal Studies

A limited number of studies have examined the effects of RE administration on tumor growth in animals in vivo (Table 6). Administration of RE (1 mg/mL) in the drinking water ad libitum for 32–35 days resulted in a significant decrease in tumor size in nude mice xenografted with SW620 colon cancer cells [21]. A similar study using HCT116 colon cancer xenografted athymic nude mice fed 100 mg/kg/day RE dissolved in olive oil (4 weeks) significantly decreased tumor size in treated animals compared to control [23]. Biochemical analysis of serum samples collected from Sprague Dawley rats with N-methylnitrosourea-induced colon cancer showed significant anticancer effects by both high (3333.3 mg/kg/day) and low (1666.6 mg/kg/day) dose RE after 4 months of treatment with significant alteration of gene and protein signaling and aggregation of lymphoid cells [43]. A significant reduction in tumor volume was seen in mice xenografted with 22RV1 prostate cancer cells by RE (100 mg/kg/day) which was administered, dissolved in olive oil for 22 days [33].

In a diethylnitrosamine (DEN)-induced liver cancer model in F344 rats, RE at 100 mg/kg/day (5 days) was administered intragastrically with an intraperitoneal (i.p) injection of DEN on day 4. From this point, rats were fed a normal diet for 3 weeks until undergoing partial hepatectomy. Examination of liver tissue suggested RE may exert some protective antioxidant effects [44]. In accordance with this, use of Swiss mice exposed to 6 Grays (Gy) ionizing radiation (IR) in their liver once, followed by treatment with 1000 mg/kg RE fed orally, daily for 5 days suggested protective, antioxidant activity by RE. A delayed onset of IR-induced mortality and attenuated increases in glycogen and protein levels were seen in livers of mice exposed to IR and fed RE, compared to IR-exposed mice not fed RE [45]. Caution should be taken however, due to the high concentration (1000 mg/kg) used [45] which is at least 10 times greater than what has been found to exert potent anticancer effects in other studies. Taken together, these studies suggest a role for RE inhibiting chemical- or IR-induced carcinogenesis by exerting protective, antioxidant effects on healthy tissues. Thus, RE may display radio-protective effects, which would benefit healthy tissue during radiation treatment.

In WEHI-3BD myeloid leukemia xenografted mice fed 1% *w*/*w* RE in their food ad libitum (29 days), investigators noted a significant decrease in both tumor volume and incidence. Furthermore, RE showed an additive effect when combined with Vitamin D analogues (VDA) [41]. In WEHI-3BD xenografted mice administered RE (4% *w*/*w* in food) for up to 15 weeks combined with VDAs, median survival time was significantly increased and white blood cell count decreased to levels comparable to those seen in the control group of healthy mice [40].

Using a 7,12-dimethylbenz(a)anthracene (DMBA)-induced skin cancer nude mouse model, RE (500 or 1000 mg/kg/day; 15 weeks) administered orally in water resulted in a significant decrease in tumor number, diameter, weight and decrease in tumor incidence and burden, and an increase in latency period compared to control mice treated with DMBA only [46,47]. One group of mice, which were administered RE for 7 days prior to the first application of DMBA, showed a 50% reduction in tumor growth compared to the DMBA-only treated mice, suggesting potent chemo protective effects [47].

## 4. Mechanisms of Anticancer Effects of Rosemary Extract (RE): In Vitro Studies

Many studies have examined the anti-proliferative and colony forming abilities of RE in vitro in colon [15,16,17,18,19,20,24,25,26], pancreas [27], breast [18,29,31,32], prostate [33], cervical [18,35], bladder [31], ovarian [34], lung [39] and leukemia [29,40,41,42] cell lines however, little is known concerning the underlying mechanism. RE was shown to have an inhibitory effect on AKT1 mRNA and protein expression, a protein involved in the PI3K/Akt survival signaling pathway, in a leukemic cell line [42] however, no measure of Akt activity was mentioned. No effect on ERK2 protein levels, involved in cell proliferation and differentiation, were seen in these cells. Cell cycle arrest prevents further division by proliferating cells and RE was shown to induce cell cycle arrest in a number of cancer cell lines [17,19,24,25,33,34,41] and increase retinoblastoma-related gene 2 (Rb2) [42] which regulates entry into cell division. Recently, Moore, et al. (2016) found RE inhibited activation of the Akt/mTOR/p70S6K signaling pathway which was associated with a significant decrease in cell proliferation and survival [39].

The viability of various cancer cell lines was shown to be significantly inhibited by treatment with RE which many studies attributed to enhanced apoptosis and cell death. Increased poly ADP ribose polymerase (PARP) cleavage, which is an established indicator of enhanced apoptosis, was seen in colon [20,21], pancreas [21], breast [32] and lung [39] cancer cell lines following treatments with RE. Alternatively, RE enhanced nitrate accumulation (i.e., increased nitric oxide production) and TNFα production in pancreatic [27] and liver [37] cancer cells, indicative of enhanced cell death capabilities and nitric oxide-induced apoptosis. In ovarian cancer cells [34] enhanced apoptosis was associated with increased gene expression of mitochondrial-regulated apoptosis proteins cytochrome c, involved in the electron transport chain, and heat shock protein 70 (hsp70) which is involved in protein folding and protecting the cell from heat stress and toxic chemicals. Other mechanisms of apoptosis by RE include enhanced protein expression of pro-apoptotic Bax and cleaved-caspase 3 [23,33], increased expression of binding immunoglobulin protein (BiP) and CCAAT/-enhancer-binding protein homologous protein (CHOP) proteins which induce endoplasmic reticular stress [25,33], and the unfolded protein response [24,25,26,33] in prostate and colon cancer cells. Interestingly, in normal prostate epithelial cells RE treatment resulted in a decrease in endoplasmic reticular stress related protein PRKR-like endoplasmic reticulum kinase (PERK), suggesting RE selectively induces endoplasmic reticular stress in prostate cancer cells but spares normal prostate cells [33]. Similarly, in breast cancer cells [32] RE decreased expression of estrogen receptor α (ERα) in the ER+ subtype and human epidermal growth factor receptor 2 (HER2) in the HER2+ subtype, and it was suggested the decreased receptor expression was correlated with enhanced apoptosis in these cell subtypes. Correspondingly, increased levels of Fos, an oncogenic transcription factor, were detected in ER+ and HER2+ cell lines, and this event is thought to precede apoptosis and correspond to the PARP-cleavage seen in these cells. Although RE was also capable of inducing anticancer effects in triple negative (TN) breast cancer cells, its mechanism has yet to be elucidated [32].

Induction of apoptosis by endoplasmic reticular stress has been found by several studies in colon cancer cells [19,23,24,26] and has been shown to involve translocation of nuclear factor erythroid 2-related factor 2 (Nrf2) into the nucleus and induction of p38 MAPK and PERK activity. The Nrf2/antioxidant response element (ARE) signaling pathway has been considered to protect cells against carcinogenesis and attenuate cancer development by neutralizing ROS and carcinogens and members of this pathway, including sestrin-2 and heme oxygenase-1 (HO-1), are upregulated by RE in colon cancer cells [23,25]. Overall, the majority of existing studies indicate that the anticancer effects of RE may be due largely to induction of apoptosis.

Antioxidants are molecules, which scavenge harmful free radicals, protecting cells from oxidative DNA damage and potentially death. RE has been shown to exert antioxidant effects in colon [15], breast [28], and leukemia [29,40] cell lines. Colon cancer cells pretreated with RE followed by treatment with hydrogen peroxide, often used in cell culture to induce oxidative DNA damage, showed reduced DNA double-strand breaks and oxidative damage compared to control cells treated with hydrogen peroxide only. Similarly, RE reduced oxidative damage induced by methylene blue (oxidizes purines) in these cells [15]. RE treatment resulted in increased levels of antioxidants and NAPD(H)-quinone reductase (oxidoreductase involved in the transfer of electrons from a reduced molecule to an oxidized molecule) which decreased reactive oxygen species (ROS) levels, and inhibited lipopolysaccharide (LPS)-stimulated production of the free radical nitric oxide (NO) in leukemia cell lines [29,40]. In an in vitro model of cigarette smoking, the use of an RE containing cigarette filter considerably reduced benzopyrene (carcinogen) levels and associated DNA adduct formation in breast cancer cells [28]. An effect of RE treatment, to inhibit ROS levels in cancer cells, may be viewed as a beneficial and not an anticancer effect for cancer cells. Traditionally treatments for cancer should result in apoptosis/killing of cancer cells. The antioxidant properties exerted by RE treatment indicate a potential for RE as a preventative strategy which may target the initiation and promotion stages of cancer. Antioxidants work to restore damaged DNA back to normal and protect the cell from further damage thus, preventing the potential mutation into a cancer cell and subsequent tumor formation.

In addition to the antiproliferative, apoptotic and antioxidant mechanisms noted above, some evidence indicates that RE may (i) exert anti-inflammatory effects [29] through inhibition of interleukin-1 (IL-1) and cyclooxygenase 2 (COX2) molecules; (ii) aid in the reversal of acquired drug resistance [34] by inhibiting P-glycoprotein levels (involved in drug resistance); and (iii) alter metabolic-related genes [21] such as glycosyltransferase (GCNT3) which forms glycosidic linkages in a variety of macromolecules and its potential epigenetic regulator microRNA-15b. Induction of autophagy [26] and alterations to cholesterol metabolism [24] may also be mechanisms of RE in colon cancer cells.

## 5. Mechanisms of Anticancer Effects of Rosemary Extract (RE): In Vivo Animal Studies

Limited evidence exists regarding RE’s mechanism in vivo however, few studies list potential antioxidant effects and serum biomarkers for RE’s anticancer effects. Increases in glutathione (GSH), an antioxidant, and reductions in lipid peroxidase (LPx), an oxidizing agent resulting in free radical production and cell damage, have been recorded in IR-induced mouse liver [45] and DMBA-induced mouse skin cancer [46,47] models treated with RE. Similarly, RE decreased glutathione-S transferase (GST) positive foci, which are associated with oxidative damage from the reduction of GSH [40], in a rat DEN-induced liver cancer model however, results were not significant and should be taken with caution [44].

Serum samples from mice xenografted with prostate cancer cells and fed RE in their diet showed a decrease in prostate-specific antigen (PSA) levels (high levels would be suggestive of prostate cancer) and examination of tissue samples showed decreased androgen receptor and CHOP expression, indicative of an induction of apoptosis associated with endoplasmic reticular stress [33]. Similarly, HCT116 colon cancer xenografted mice showed increased Nrf2 and Sestrin-2 expression which are indicative of endoplasmic reticular stress and can lead to enhanced apoptosis [23]. A significant decrease in microRNA-15b (miR-15b) plasma levels after administration of RE in colon cancer xenografted mice suggested circulating miR-15b levels may act as a minimally invasive method to monitor the antitumor effects of RE in vivo [21]. Furthermore, rats with *N*-methylnitrosourea (NMN)-induced colon cancer fed RE, showed significant alterations in cell death modulating proteins including cytochrome c, programmed cell death protein 4 (PCDP4), carcinoembryonic antigen (CEA) and colon-cancer specific antigen-4 (CCSA-4) [43]. Sufficient evidence exists to support the potential use of RE in chemotherapeutics however, it is still not well understood whether the anticancer effects seen by RE are attributable to individual polyphenols within the extract or rely on the combination of all the components within the extract combined. The next section of this review explores the role of two of RE’s main polyphenols, CA and RA, and their potential contribution to RE’s anticancer effects.

## 6. Anticancer Effects of Carnosic Acid (CA): In Vitro Studies

Treatment of different colon cancer cells with CA resulted in significant inhibition of cell viability using concentrations ranging from 1 to 400 µM, and having IC50 values in the 20–90 µM range (Table 7). In addition, CA induced apoptosis and cell cycle arrest in Caco-2 cells [48,49] and inhibited cell adhesion and migration in Caco-2, HT-29 and LoVo cells [49] by inhibiting activity of the cell cycle regulator cyclin A [48] and by inhibiting MMP-9, uPA and COX-2 activity, associated with cell adhesion and migration properties [49]. Similarly, in SW480 colorectal cancer cells with hyperactive β-catenin which is oncogenic, CA targeted β-catenin for proteasomal degradation and this suggests a potential for CA to be used as a small molecule oncogenic β-catenin inhibitor [50]. In SLW620 and DLD-1 cells CA inhibited cell viability and this was associated with downregulation of miR-15b and enhanced GCNT3 activity which are associated with regulation of metabolic related genes [21]. Furthermore, in HT-29 colon cells CA inhibited cell proliferation and enhanced cell cycle arrest, which was correlated with altered expression of an array of transport and biosynthesis genes and altered activity of detoxifying enzymes and metabolites. Of note, levels of GSH, an important antioxidant, were enhanced and levels of *N*-acetylputrescine, which are toxic in high doses, were decreased [51]. In HT-29 cells co-cultured with 3T3-L1 adipocytes, CA attenuated the negative effects of the adipocytes on the colon cancer cells by inhibiting triglyceride accumulation and downregulating expression of the Ob-R receptor [52]. In these cells CA also inhibited cell viability by decreasing phosphorylation of the cell survival regulators Akt and Bcl-xL and enhancing Bax expression. Furthermore, cell cycle arrest was induced by inhibition of cyclin D1 and CDK4 [52]. Similarly, a fraction of rosemary extract which was found to consist mainly of CA (98.7% pure) was tested on HT-29, SW480 and HGUE-C-1 colon cancer cells and significantly inhibited cell viability. Among several different fractions of the RE that were tested, the fraction containing CA was found to be among the most active and it was suggested that synergism between many components of the extract plays a role in rosemary’s anticancer effects [22]. Inhibition of cell proliferation and increased cell cycle arrest by CA in HT-29 cells was found to be orchestrated by the unfolded protein response and triggered by endoplasmic reticular stress [24] which can lead to apoptosis and thus destruction of cancerous cells. Enhanced cholesterol and ROS accumulation in CA treated cancer cells was also shown to contribute to the inhibition of proliferation seen [24]. Similarly, activity of pro-apoptotic markers including p53, Bax, caspases and PARP were enhanced and anti-apoptotic markers MDM2, Bcl-2 and Bcl-xL were decreased in HT-29, HCT116 and SW480 colon cells [53]. Levels of ROS and H_2_O_2_ were increased in vitro in the cell medium [25,53] by CA which can trigger cellular stress and thus cancer cell death. The signaling molecules STAT3 and survivin play a key role in regulating cell survival and CA inhibited activity of these molecules in colon cancer cells [53]. These studies provide strong evidence that CA at relatively low doses (1–100 µM) is capable of inhibiting colon cancer cell growth and survival by modulating expression of key signaling molecules and altering cell metabolism.

In breast cancer cells, including MCF-7, MDA-MB-231 and MDA-MB-468, CA inhibited cell proliferation and enhanced apoptosis at concentrations of 1.5–150 µM [30,54,55,56] (Table 8). The inhibitory effects of CA were found to be dependent on increasing levels of the antioxidant glutathione in breast cancer cells and accordingly, expression of genes involved in glutathione biosynthesis (CYP4F3, GCLC) and transport (SLC7A11) were significantly increased as well [54]. Importantly, the sensitivity of CA was found to be associated with HER2 expression and thus the MCF-7 cells were more sensitive to the CA treatment, compared to the triple-negative MDA-MB-468 cell line which does not express HER2 [54]. In the triple negative MDA-MB-361 cell line CA induced TRAIL-mediated apoptosis through down-regulation of c-FLIP and Bcl-2 expression and through CHOP-dependent upregulation of DR5, Bim and PUMA expression (ER stress associated proteins) [56] suggesting that CA is capable of inhibiting breast cancer cell survival through different mechanisms depending on the mutations that are present.

Inhibition of cell viability by CA was shown in rat insulinoma (RINm5F) and human (MIA-PaCa-2, PANC-1) pancreatic cancer cells at doses of 6–300 µM [21,27]. In prostate cancer cells, lower doses of CA (<100 µM) inhibited cell viability and enhanced apoptosis [30,57,58]. Induction of apoptosis in PC-3 prostate cells was associated with activation of both intrinsic and extrinsic apoptotic pathways. Inhibition of caspase 8 and 9, Bcl-2, Bid, IAP, p-Akt, p-GSK3 and NF-κB and activation of caspase 3 and 7, PARP, Bax, cytochrome c and PP2A all contribute to enhanced apoptosis within these cells [58]. The use of a pan-caspase inhibitor attenuated the apoptotic effects of CA and provides strong evidence for the involvement of caspases in the apoptotic mechanism of CA in prostate cancer cells [58]. Low doses of CA both alone and in combination with other phytonutrients such as curcumin showed potent anticancer effects in LNCaP, PC3 and DU145 prostate cells and inhibited androgen receptor activity. The inhibition of proliferation of these cells was associated with an inhibition of the EpRE/ARE antioxidant transcription system and inhibition of PSA secretion [57]. Furthermore, CA inhibited proliferation of A2780 ovarian cancer cells and enhanced the sensitivity of a resistant A2780CP70 cell line to cisplatin, a potent chemotherapeutic agent [34]. Carnosic acid has potent anticancer effects on its own but also acts synergistically with other compounds including phytonutrients and chemotherapeutics and this represents a promising route for future cancer therapies using combinations of anticancer agents at lower doses.

In Hep-3B, HepG2 and SK-HEP1 human liver cancer cells, CA inhibited cell viability and enhanced apoptosis [30,55,56,59] (Table 9). In Hep-G2 cells the formation of autophagic vacuoles and autolysosomes contributed to enhanced cell death by CA and this was induced through inhibition of the Akt/mTOR cell survival pathway [59]. Furthermore, in SK-HEP1 cells CA induced TRAIL-mediated apoptosis by altering apoptotic markers such as c-FLIP, Bcl-2, DR5, Bim, PUMA and CHOP [56]. Rat liver clone 9 cells are often used as a model for screening hepatotoxicity and CA was found to enhance activity of enhancer element GPEI which regulates the pi class of glutathione *S*-transferase and modulates antioxidant and detoxification systems within the cell [60]. CA was found to exert a protective effect in these non-cancerous liver cells which was modulated by the Nrf2/p38 MAPK signaling pathway [60,61]. Furthermore, CA inhibited viability of small-cell lung cancer NCI-H82 cells [30].

In several models of skin cancer, including HT-1080, BEAC, HUVEC and B16F10 cells, CA inhibited cell survival, cell migration and cell adhesion, enhanced apoptosis and induced cell cycle arrest [62,63] (Table 9). Chromatin condensation and DNA fragmentation were seen in HT-1080 cells which lead to apoptosis [62]. In human umbilical and bovine aortic endothelial cell lines, CA inhibited tubulogenesis and MMP-2 expression suggesting anti-angiogenic properties of CA which would be beneficial in anticancer therapies [62]. Inhibition of the epithelial-mesenchymal transition in B16F10 melanoma cells suggests a possible mechanism for the inhibition of cell migration by CA. Inhibition of cell migration markers MMP-9, TIMP-1, uPA and VCAM-1 was seen in this cell line using low doses of CA (10 µM). Inhibition of phosphorylation of signaling molecules Akt, FAK and Src were also associated with inhibition of the epithelial-mesenchymal transition and cell migration in B16F10 cells [63]. In Caki, kidney cancer cells, CA induced apoptosis through ROS-mediated endoplasmic reticular stress. Activity of apoptotic markers PARP, caspase 3, ATF4 and CHOP was increased in these cells [55]. Similarly, TRAIL-mediated apoptosis was induced in Caki, AHCN and A498 kidney cells through modulation of endoplasmic reticular stress related proteins c-FLIP, Bcl-2, DR5, Bim, PUMA and CHOP [56].

In T98G glioblastoma cells CA promotes production of nerve growth factor and this was found to be regulated by the Nrf2 signaling pathway [64,65] (Table 10). Nerve growth factor is involved in the regulation of growth and the maintenance and survival of certain target neurons, and thus can act to protect neural cells from toxic agents that may cause cancer. In IMR-32 neuroblastoma cells CA induced apoptosis by activation of caspases, PARP and the p38 MAPK pathway and inhibited cell viability, which was associated with decreased ERK activation [66]. Interestingly however, in SH-SY5Y neuroblastoma cells CA attenuated apoptosis induced by the neurotoxic compounds methylglyoxal and amyloid β, exerting a cytoprotective effect [67,68]. This protective effect was associated with increased activation of PI3K/Akt signaling, inhibition of cytochrome c release and inhibition of caspase cascades which results in a pro-survival effect on the cell [36,67]. Similarly, in U373MG astrocytoma cells CA inhibited amyloid β peptide production and release and this was associated, at least partially, with activation of the α-secretase TACE/ADAM17 [69]. The use of CA may have potential in the prevention of amyloid β-mediated diseases. Furthermore, in GBM glioblastoma cells, CA promoted apoptosis by inducing cell cycle arrest and degradation of cyclin B1, RB, SOx2 and GFAP, molecules involved in cell survival and maturation processes [70].

Leukemia is a cancer that usually develops in the bone marrow and results in a high number of white blood cells that are not fully developed being released into the bloodstream. Most treatment options for leukemia involve agents that promote the differentiation of these immature white blood cells into mature, differentiated cells. Unfortunately, there are many side effects associated with higher doses of these differentiating agents and strategies are required to lower the dose necessary to see anticancer effects. One such agent which is used is 1α25-dihydroxyviatmin D (1,25D). Many studies have found that low doses of CA (5–10 µM) are able to potentiate the pro-differentiation effects of 1,25D and help sensitize leukemia cells including human HL-60, U937, MOLM-13 and mouse WEHI-3B cells [40,41,71,72,73,74,75,76,77,78] to its anticancer effects (Table 11). Furthermore, CA inhibited cell viability and induced apoptosis and cell cycle arrest in these cells using a multitude of different strategies. In HL-60 cells, CA enhanced expression of the vitamin D and retinoic acid receptors thus, enhancing the sensitivity of cells to 1,25D [71], and enhanced expression of cell cycle regulators p21^Waf1^, p27^Kip1^ which may have tumor suppressor functions [72]. Carnosic acid also increased levels of the antioxidant GSH and phase II enzyme NADP(H)-quinone reductase which help protect cells from chemically-induced carcinogenesis, and enhanced signaling through MAPK pathways including ERK and JNK which are involved in the proliferation and differentiation of cells [40,73,74,75,79]. In K562 leukemia cells CA inhibited cell viability and sensitized resistant cells to adriamycin, a chemotherapeutic agent [30,80]. Similarly, CA enhanced the activity of doxercaliferol, an agent which helps prevent the common problem of calcification associated with administration of vitamin D derivatives such as 1,25D, and decreased levels of microRNA181a which are linked to cell proliferation [81]. Antioxidant effects were also produced by CA in U937, HL-60 and NB4 leukemic cells which exhibited increased GSH and NADPH levels and CA ameliorated arsenic trioxide-induced cytotoxic effects [79]. Activation of the Nrf2/ARE signalling pathway which can alter cell survival was also seen [77,79]. The authors suggest that the Nrf2/ARE pathway likely plays an important role in the cooperative induction of leukemia cell differentiation by 1,25D and CA [77]. Importantly, in HL-60 cells CA increased PTEN expression and caspase cleavage and inhibited phosphorylation of Bad and Akt which are associated with enhanced apoptosis [62,82]. The strong inhibitory effects of CA on the PTEN/Akt survival pathway make it a good candidate to be combined with other therapies for leukemia treatment.

## 7. Anticancer Effects of Carnosic Acid (CA): In Vivo Animal Studies

The above studies in vitro provide strong evidence for the anticancer effects if CA in various cancer cell lines. Several studies using animal models have also explored the effects of CA in vivo and found significant anticancer effects which supports future research exploring the anticancer mechanisms of CA in both animal and human models (Table 12). In DMBA-induced models of oral cancer using hamsters, it was shown that using 10 mg/kg/day CA administered orally for 14 weeks, caused the number of tumors on the animals to significantly decrease. Furthermore, expression of detoxification enzymes was enhanced [83], markers of apoptosis including p53, Bax, Bcl-2 and caspases were increased [84], and regulators of cell growth including COX-2, c-fos, KF-κB and cyclin D1 were decreased [84]. Using the same hamster model, 750 µg CA dissolved in 0.1mL saline (20 µM) administered daily for 11 weeks significantly slowed the progression of lesions and oral cancer development [85]. In mice xenografted with prostate samples from human biopsies, 100 mg CA dissolved in 100 µL of cottonseed oil administered daily for 25 days decreased tumor growth [86]. Azoxymethane was used to induce colon cancer in mice and 0.01%–0.02% CA fed with a high fat (45%) diet for 11 weeks decreased both tumor size and number of tumors, and modulated signaling molecules involved in cell metabolism and cell growth [52]. Serum samples taken from the mice after treatment showed decreased levels of insulin, leptin and IGF-1 and analysis of tissue samples showed a decrease in the associated insulin and leptin receptors, as well as decreased activity of ERK and expression of cyclin D1 and Bcl-xL which regulate cell survival [52]. In K562 leukemia inoculated mice fed 1% CA with standard powder diet, there was a decrease in the number of leukemic cells which was partially attributed to enhanced apoptosis [87]. Furthermore, survival time of the animals increased significantly [87]. Overall, CA shows significant anticancer effects in mouse and hamster models of several types of cancer and this evidence provide support of its potential to be used against cancer in humans.

## 8. Anticancer Effects of Rosmarinic Acid (RA): In Vitro Studies

Treatment of HT29 colon cancer cells with RA (5–20 μM) lead to a reduction in COX2 promoter activity and COX2 protein levels [88] (Table 13). In HCT15 and CO115 colon cancer cells, RA (10–100 μM) induced apoptosis and decreased levels of phosphorylated-ERK which regulates cell proliferation [89]. Rosmarinic acid (55–832.6 µM) decreased ROS levels which was associated with decreased migration and adhesion rates in Ls174-T colon cells [90]. Furthermore, treatment of CO115 cells with RA (50 μM) protected against BCNU-induced DNA damage, suggesting potential chemopreventive effects [91]. Treatment of MCF-7 and MDA-MB-231 breast cancer cells with RA (0–300 μM) decreased cell viability [30,92,93,94] (Table 13). Rosmarinic acid decreased methyltransferase activity, which inhibits hyper-methylation of DNA, associated with disease [93], and sensitized a resistant cell line (MCF-7/Adr) to the chemotherapeutic agent Adriamycin [94].

In DU145 and PC3 prostate cancer cells RA (17.3–138.8 µM) decreased cell viability [30] and in A2780 and A2790CP70 ovarian cancer cells RA (6.9–27.8 µM) lead to a reduction in cell proliferation and increased the sensitivity of cisplatin-resistant cells [34] (Table 13). In SCG7901/Adr gastric cancer cells, RA (0.096–60 μM) was found to decrease cell viability, drug resistance, expression and activity of p-glycoprotein [95]. Furthermore, treatment of MKN45 gastric cancer cells with RA (200–300 μM) lead to a decrease in cell viability, the Warburg effect/glucose uptake and pro-inflammatory cytokines [96]. In B16 melanoma cells, RA (1–100 μM) was found to increase melanin content, tyrosinase expression and CREB phosphorylation [97].

Treatment of HepG2 liver cancer cells with RA (25–250 μM) decreased ochratoxin and aflatoxin-mediated cell damage, apoptosis, ROS levels and caspase 3 activation [98] (Table 14), suggesting that RA can exert protective effects and prevent cytotoxicity induced by toxic agents. Alternatively, in HepG2 cells without the presence of cytotoxic agents, RA (13.9 and 27.8 µM) lead to an increase in apoptosis, which was associated with an increase in caspase 8, NFBIA, TNFSF9 and Jun mRNA and a decrease in Bcl-2 mRNA levels [99]. Thus, RA has several potential anticancer mechanisms in liver cells. In Hep-3B liver cancer cells, RA (17.3–138.8 µM) was found to decrease cell viability [30], while treatment of HepG2 liver cancer cells with RA (20–80 μM) showed no significant changes to cell viability but an increase in Nrf2 nuclear translocation, ARE-luciferin activity, MRP2 levels, intracellular ATP levels and efflux of p-glycoprotein was seen [100]. In NCI-H82 and A549 lung cancer cells RA (10–500 µM) decreased cell growth [30,101] which was associated with decreased hCOX2 activity, suggesting an anti-inflammatory role of RA [101].

Treatment of K562 leukemia cells with RA inhibited cell viability [30] and reversed the induction of hyperosmosis-induced apoptosis and associated ROS/RNS production [102] (Table 15). In U937 leukemia cells, RA (60 μM) enhanced TNF-α induced apoptosis and decreased TNF-α induced-NF-κB activation and ROS production [92]. Surprisingly AKT1 and ERK2 levels, which regulate cell survival, were not affected by RA treatment in U937 or K562 cells [42]. Rosmarinic acid (40 μM) increased macrophage differentiation induced by ATRA which was mediated by an increase in CD11b expression on the cell surface [103]. In HL-60 leukemia cells, RA (50–150 μM) inhibited cell growth and induced apoptosis, which was associated with decreased dNTP levels [104]. CCRF-CEM, CEM/ADR5000 leukemia cells treated with RA (3–100 μM) developed increased cytotoxicity, apoptosis, necrosis, cell cycle arrest and caspase-independent apoptosis which was mediated by increased PARP cleavage and blockage of p65 nuclear translocation [105]. In agreement with other studies, RA (0.07–2.2 mM) exerted DNA protective and anti-carcinogenic effects in HL-60 leukemia cells [106].

## 9. Anticancer Effects of Rosmarinic Acid (RA): In Vivo Animal Studies

Apart from the in vitro studies using different cancer cell lines, several studies using RA in animal cancer models have been performed. Administration of 0.25–1.35 mg of RA (30 min) prior to TPA treatment was found to decrease myeloperoxidase activity and COX2 induction in mice [107] (Table 16). Using 1–4 mg/kg RA (20 days) in Lewis lung carcinoma xenografted mice lead to decreased tumor growth [90] and 100 mg/kg RA (14 weeks) reduced DMBA-induced tumor formation in the buccal pouches of hamsters [108]. Administration of 360 mg/kg RA from weeks 4 to 12 of the animal’s life decreased the frequency of large adenomas in mice [109]. Rats given 2.5–10 mg/kg RA for 16 weeks, showed a decrease in development of DMH-induced aberrant crypt foci by decreasing DMH-induced elevation of bacterial enzymes [110]. Administration of 100 mg/kg RA 1 week before DMBA treatment in mice decreased skin tumors by increasing the levels of phase I (cyt p450) and phase II (GST, GR, GSH) detoxification agents and restoring levels of caspase 3, caspase 9, p53 and Bcl-2 [111]. Venkatachalam, et al. found that 2.5, 5 and 10 mg/kg RA given to rats for 4 weeks, decreased DMH-induced colon tumor formation, number of polyps, antioxidant status, CYP450 content, PNPH activity and reversed the markers of oxidative stress [112]. Hamsters given 1.3 mg/mL RA for 2 weeks were found to have a decreased incidence of tumors induced by DMBA, decreased tumor grade scoring and increased tumor differentiation [113]. Rosmarinic acid administered at 2 mg/kg for 14 days to mice had an anti-Warburg effect, mediated through decreased glucose uptake [96]. Furthermore, administration of 5 mg/kg RA for 30 weeks was found to decrease DMH-induced colon tumor formation in rats through decreased TNF-α, IL-6 and COX2 levels [110]. Taken together, these studies provide evidence for RA’s anticancer effects in animal models and suggest several mechanisms which may be responsible for the inhibition of tumor growth and progression.

## 10. Dosage and Bioavailability

The effects of RE have been studied in many cancer cell lines and although the concentrations used in the in vitro studies are variable (0.1–500 μg/mL) it appears that the concentrations in the range of 0.1–100 μg/mL are most effective. Similar to in vitro studies, the reported doses of RE used in vivo are within a wide range (1 mg/mL drinking water −3333.3 mg/kg/day). This high variability suggests the need for more systematic studies to identify effective RE doses in vivo. One study has examined the levels of RE components in the plasma and tissue samples of animals administered with RE. Administration of a single dose of RE (100 mg/mL water) enriched in CA (40% *w*/*w*) by intragastric gavage in rats was followed by measurements of RE compounds and metabolites in plasma, liver, small intestine content and brain. The researchers tentatively identified 26 compounds and the main metabolites detected in plasma, liver and gut were glucuronide conjugates of CA, carnosol and rosmanol [115]. Metabolites were detected as early as 25 min after oral administration and most of the compounds remained present at substantial concentrations (micromolar range) for several hours [115]. Doolaege, et al. reported that 64.3 mg/kg (193.43 mM) CA orally administered to rats resulted in a plasma concentration of 0.015 mg/mL (45.12 μM) [116]. Another study reported that ingestion of 360 mg/kg/day RA after 8 weeks resulted in a plasma concentration of 1.1 μM [117]. The reported plasma concentrations of CA, carnosol and their metabolites were in the micromolar range indicating that absorption and bioavailability are likely not barriers for these components of RE [114,115,118].

Another important issue that must be systematically examined in well-designed studies are the potential toxicity of chronic administration of RE and RE polyphenols. Rosemary extract has already been approved as a safe food additive by the European Food and Safety Authority (EFSA) [119] and is considered to be generally recognized as safe by the United States Food and Drug Administration (FDA) (21CFR182.10). In a study reviewed by the EFSA, rosemary was found to have low acute and sub-chronic toxicity in rats and the only effect at high doses was a slight increase in relative liver weight, which has been shown to be reversible. Overall, 90 day RE administration (180–400 mg/kg/day, equivalent to 20–60 mg/kg/day of carnosol plus CA) in rats revealed no observed adverse effect levels (NOAEL) (reviewed in [119]). Furthermore, an acute single dose of 24 and 28.5 g/kg RE to female and male mice respectively or the daily administration of 11.8 and 14.1 g/kg to female and male mice respectively for 5 days resulted in no gross macroscopic lesions observed on autopsy besides fatty liver in mice subjected to repeat administration of the extract indicating low acute toxicity (reviewed in [119]). In another study, it was reported that an LD50 of 169.9 mg/kg/day RA was found in mice implanted with Lewis lung carcinoma cells [90]. One study performed in humans used a powdered RE mixed with citrus extract (1:1 ratio) (Nutroxsun™) which was consumed daily (250 mg) for 3 months. Results showed a protective effect against UV-induced skin damage. Significant results were seen after 8 weeks and continued to increase after 85 days of treatment [120]. Overall, the limited in vivo studies report doses of RE or RE components that are relatively high and showed minimal to no adverse effects, indicating low toxicity. Nonetheless, further research should be performed to confirm maximum recommended doses of RE and RE components.

In humans, to achieve RE polyphenol levels that will provide health benefits high intake of rosemary would be required, which is not practical. A more reasonable direction for the potential future use of RE and its polyphenols as anticancer agents would be to develop easily ingestible and soluble pills containing RE or RE components. Overall, the studies available currently suggest that RE and its polyphenols CA and RA are good candidates for drug development and further research examining the effective doses in animals is required before any clinical studies in humans are initiated. In addition, systematic studies in animals to examine if chronic administration results in any toxicity are required before clinical human studies.

It should be noted that in recent years, scientists have recognized that the gut microbiota plays an important role in overall health and disease prevention. Although certain plant bioactive compounds may be poorly bioavailable, the gut bacteria may generate metabolites that are more potent than the parent compounds. A recent study found that administration of RE rich in CA (40% *w*/*w*) in rats had a selective effect on caecum microbiota (increased the Blautia coccoides and Bacteroides/Prevotella groups and reduced the Lactobacillus/Leuconostoc/Pediococccus group), decreased β-glucosidase activity and increased fiber fecal elimination [121]. These data are associated with the decreased body weight and the improvement of the metabolic and inflammatory status seen with RE [121]. Although the above study suggests a potential prebiotic effect of RE administration against metabolic disorders and obesity, there are no studies specifically examining the effect of gut microbiota on RE metabolites.

## 11. Conclusions

It should be noted that the levels of polyphenols and bioactive compounds present in RE may be affected by many factors such as the plant growing conditions (soil, climate, exposure to stressors). Additionally, the extraction method and storage of RE may affect its potency. Water, methanol, ethanol and supercritical carbon dioxide extraction are methods which have been used in different studies and evidence suggests that methanol (alcoholic-solvent) extraction may lead to RE with higher potency (lower IC50) [31]. Since the source and extraction method of RE may affect its potency/biological activity, this issue should be taken into consideration when future studies are planned.

In recent years, focus has shifted towards establishing new targeted cancer treatments that can modulate specific pathways often mutated in cancer. RE and its polyphenols CA and RA may be used as chemicals to target specific pathways leading to induction of apoptosis and decreased cell survival. In addition, RE, CA and RA may be used as neutraceuticals to enhance the anticancer effects of current chemotherapeutics. This could allow for lower doses to be used and less toxicity induced in healthy surrounding tissue. Although studies examining signalling molecules and pathways targeted by RE, CA and RA are limited, the existing studies provide supporting evidence for the use of these compounds both on their own and in combination with other cancer therapies.

Overall, RE, CA and RA have been shown to have various potent and effective anticancer properties. However, more systematic studies are required in animals before human studies are initiated. The in vivo animal studies should find (1) the doses to be administered; (2) the best route of administration; (3) the plasma levels of CA, RA and other RE bioactive ingredients; (4) the signaling molecules/pathways affected; and (5) any possible toxic effects associated with chronic administration.

## Figures and Tables

**Table 1:** Anticancer effects of Rosemary Extract (RE). In vitro studies: colon cancer.

Cancer Cell	Dose/Duration	Findings	Mechanism	Reference
CaCo-2 (Colorectal adenocarcinoma)	0.1–30 µg/mL (3–24 h)	↓ cell colony formation. Long and short term antioxidant effects	↓ H_2_O_2_-induced DNA strand breaks and oxidative damage. ↓ visible light-induced oxidative damage	[15]
SW480 (Colorectal adenocarcinoma)	31.25–500 µg/mL (48 h)	↓ cell proliferation. Cytotoxic above 250 µg/mL. IC50~71.8 µg/mL		[16]
HT-29 (Colorectal adenocarcinoma)	RE containing 10 µM total polyphenols (72 h)	↓ cell proliferation ↑ cell cycle arrest ↑ apoptosis		[17]
HT29 (Colorectal adenocarcinoma)	1.95–62.5 µg/mL (48 h) 3 RE’s standardized to 25.9%, 36.2%, 42.4% CA	↓ cell proliferation IC50 > 62.5 µg/mL		[18]
SW480 (Colorectal adenocarcinoma), HT29 (Colorectal adenocarcinoma)	RE containing 10 µM total polyphenols (48 h)	↓ cell proliferation SW480 more sensitive ↑ cell cycle arrest	↑ antioxidant and xenobiotic effects Modulates: Nrf2, ER stress genes, cell cycle, proliferation genes	[19]
SW620 (Colorectal adenocarcinoma), DLD-1 (Colorectal adenocarcinoma)	20–110 µg/mL (24–48 h)	↓ cell proliferation IC50 36.4 and 34.6 µg/mL Effect on 5-FU sensitive and resistant cells ↑ apoptosis ↓ cell transformation	Modulates TYMS and TK1. ↑ PARP cleavage	[20]
SW620 (Colorectal adenocarcinoma), DLD-1 (Colorectal adenocarcinoma)	20–120 µg/mL (48 h)	↓ cell viability IC50 25 µg/mL	↑ PARP cleavage. ↑ GCNT3. ↓ miR-15b gene expression	[21]
HT-29 (Colorectal adenocarcinoma), W480 (Colorectal adenocarcinoma), HGUE-C-1 (Colorectal carcinoma)	1.5–100 µg/mL (24–48 h)	↓ cell viability		[22]
HCT116 (Colorectal carcinoma), SW480 (Colorectal adenocarcinoma)	10–100 µg/mL (24 h, 48 h, 72 h) Standardized to 23% CA	↓ cell viability ↑ apoptosis	↑ Nrf2 ↑ PERK ↑ sestrin-2 ↑ HO-1 ↑ cleaved-casp 3	[23]
HT-29 (Colorectal adenocarcinoma)	30 µg/mL (2–72 h)	↓ cell proliferation ↑ cell cycle arrest ↑ cholesterol accumulation ↑ ROS accumulation	↑ UPR ↑ ER-stress ↓ cell cycle genes Altered cholesterol-modulating genes	[24]
HT-29 (Colorectal adenocarcinoma)	30–70 µg/mL (24 h, 48 h)	↓ cell proliferation IC50 16.2 µg/mL ↑ necrosis	↑ Nrf2 pathway ↑ UPR ↑ autophagy	[25]
HT-29 (Colorectal adenocarcinoma)	30–60 µg/mL (6 h, 24 h)	↓ cell proliferation ↑ cell cycle arrest	↑ H_2_O_2_ in media ↑ ROS levels ↑ HO-1 and CHOP expression	[26]

H_2_O_2_ (hydrogen peroxide), 5-FU (fluorouracil), TYMS (thymidylate synthase), TK1 (thymidine kinase 1), PARP (poly ADP ribose polymerase), GCNT3 (glucosaminyl (*N*-acetyl) transferase 3), miR-15b (microRNA-15b). GI50 (50% growth inhibition), TGI (total growth inhibition), Nrf2 (nuclear factor erythroid 2-related factor 2), casp (caspase), UPR (unfolded protein response), ER (endoplasmic reticulum), HO-1 (heme oxygenase protein-1), CHOP (C/EBP homologous protein).

**Table 2:** Anticancer effects of Rosemary Extract (RE). In vitro studies: pancreatic and breast cancer.

Cancer Cell	Dose/Duration	Findings	Mechanism	Reference
RINm5F (Insulinoma)	12–100 µg/mL (24–48 h)	↓ cell proliferation ↓ cell viability ↑ apoptosis	↑ nitrate accumulation. ↑ TNFα production.	[27]
MIA-PaCa-2 (Pancreatic carcinoma), PANC-1 (Pancreatic carcinoma)	20–120 µg/mL (48 h)	↓ cell viability	↑ PARP-cleavage	[21]
MCF-7 (*ER+*) (Breast adenocarcinoma)	40 mg RE powder filter (inserted into cigarette) (2 h)		↓ BP levels and associated DNA adduct formation.	[28]
MCF-7 (*ER+*) (Breast adenocarcinoma), MDA-MB-468 (Breast adenocarcinoma)	0.1%–20% (5–120 h)	IC50 ~90 µg/mL and 26.8 µg/mL		[29]
MCF-7 (*ER+*) (Breast adenocarcinoma), MDA-MB-231 (Breast adenocarcinoma)	6.25–50 µg/mL (48 h)	↓ cell viability IC50 ~20.42 µg/mL		[30]
MCF-7 (Breast adenocarcinoma)	1–250 µg/mL (48 h)	↓ cell proliferation IC50 187 µg/mL		[31]
MCF-7 (Breast adenocarcinoma)	1.95–62.5 µg/mL (48 h) 3 REs standardized to 25.9%, 36.2%, 42.4% CA	↓ cell proliferation IC50 9.95–13.89 µg/mL		[18]
SK-BR-3 (*HER2+*) (Breast adenocarcinoma), UACC-812 (*HER2+*) (Breast ductal carcinoma), T-47D (*ER+*) (Breast ductal carcinoma), MCF-7 (*ER+*) (Breast adenocarcinoma), MDA-MB-231 (Breast adenocarcinoma)	10–120 µg/mL (48 h)	↓ cell viability Enhanced effect of chemotherapeutics ↑ apoptosis ↓ cell transformation	↑ FOS levels ↑ PARP cleavage ↓ HER2 ↓ ERBB2 ↓ ERα receptor.	[32]

TNFα (tumor necrosis factor), PARP (poly ADP ribose polymerase), BP (benzopyrene), Fos (FBJ murine osteogenic sarcoma virus), HER2 (human epidermal growth factor receptor 2), ERBB2 (HER2/neu gene), ERα (estrogen receptor α).

**Table 3:** Anticancer effects of Rosemary Extract (RE). In vitro studies: prostate, ovarian, cervical and bladder cancer.

Cancer Cell	Dose/Duration	Findings	Mechanism	Reference
DU145 (Prostate adenocarcinoma), PC3 (Prostate adenocarcinoma)	6.25–50 µg/mL (48 h)	↓ cell viability IC50 ~8.82 µg/mL		[30]
LNCaP (Prostate adenocarcinoma), 22RV1 (Prostate carcinoma)	10–50 µg/mL (24–48 h) RE standardized to 40% CA	↓ cell proliferation ↑ cell cycle arrest ↑ apoptosis modulates endoplasmic reticulum stress proteins.	↑ CHOP ↓ PSA production ↑ Bax ↑ cleaved-casp 3 ↓ androgen receptor expression	[33]
5637 (Bladder carcinoma)	0–250 µg/mL (48 h)	↓ cell proliferation IC50 48.3 µg/mL		[31]
A2780 (Ovarian carcinoma), A2780CP70 (cisplatin-resistant) (Ovarian carcinoma)	0.05%–0.25% (24–48 h)	↓ cell proliferation Enhanced sensitivity of cisplatin -resistant cell lines. ↑apoptosis ↑ cell cycle arrest Modulates expression of apoptotic genes.	↓ P-glyco protein ↑ cytochrome c gene ↑ hsp70 gene	[34]
HeLa (Cervical adenocarcinoma)	1.56–400 µg/mL (72 h)	↓ cell proliferation IC50 23.31µg/mL		[35]
HeLa (Cervical adenocarcinoma)	1.95–62.5 µg/mL (48 h) 3 REs standardized to 25.9%, 36.2%, 42.4% CA	↓ cell proliferation IC50 10.02–11.32 µg/mL		[18]
SK-OV3 (Ovarian adenocarcinoma), HO-8910 (Ovarian carcinoma)	0.0625%–1% rosemary essential oil (48 h)	↓ cell viability IC50 0.025% (SK-OV3) IC50 0.076% (HO-8910)		[36]

CHOP (C/EBP homologous protein), PSA (prostate specific antigen), Bax (Bcl-2 associated X protein), casp (caspase), hsp70 (heat shock protein 70).

**Table 4:** Anticancer effects of Rosemary Extract (RE). In vitro studies: liver and lung cancer.

Cancer Cell	Dose/Duration	Findings	Mechanism	Reference
Hep-3B (Hepatocellular carcinoma)	0.5–5 µg/mL (24 h)	↓ cell viability	↑ TNFα	[37]
Hep-3B (Hepatocellular carcinoma)	6.25–50 µg/mL (48 h)	↓ cell viability IC50 ~22.88 µg/mL		[30]
Bel-7402 (Hepatocellular carcinoma)	0.0625%–1% rosemary essential oil (48 h)	↓ cell viability IC50 0.13%		[36]
HepG2 (Hepatocellular carcinoma)	10–120 µg/mL (48 h)	↓ cell viability IC50 42 µg/mL GI50 20 µg/mL		[38]
NCI-H82 (Lung carcinoma; SCLC)	6.25–50 µg/mL (48 h)	↓ cell viability IC50 ~24.08		[30]
V79 (Normal hamster lung)	0.1–30 µg/mL (3–24 h)	Cytotoxic to cells at 30 µg/mL (24 h) Long and short term antioxidant effects	↓ H_2_O_2_-induced DNA strand breaks and oxidative damage. ↓ visible-light induced oxidative damage	[15]
A549 (Lung adenocarcinoma)	2.5–200 µg/mL (48–72 h)	↓ cell proliferation ↓ cell survival ↑ apoptosis IC50 ~15.9	↓ p-Akt ↓ p-mTOR ↓ p-P70S6K ↑ PARP cleavage	[39]

mTOR (mammalian target of rapamycin), PARP (poly(ADP-ribose) polymerase).

**Table 5:** Anticancer effects of Rosemary Extract (RE). In vitro studies: leukemia.

Cancer Cell	Dose/Duration	Findings	Mechanism	Reference
WEHI-3B D (Murine myeloid leukemia), HL-60 (Myeloid leukemia), U937 (Myeloid leukemia)	RE (10 µM equivalent of CA) (48–96 h)	Potentiated following effects of VDA: ↓ cell proliferation ↑ cell cycle arrest ↑ cell differentiation ↑ apoptosis	↑ G1 phase	[41]
RAW 264.7 (Murine leukemia; macrophage), HL-60 (Myeloid leukemia), K-562 (Human leukemia)	0.1%–20% (5–120 h) (1–200 mg/mL)	↓ cell proliferation IC50 ~18.76 µg/mL and 33.5 µg/mL ↑ cell differentiation ↓ LPS-stimulated (LS) antioxidant activity	↓ (LS) NO ↑ antioxid-ant activity ↔ basal TNFɑ, IL-1β, iNOS or COX2 ↓ (LS) IL-1β and COX2	[29]
WEHI-3B D (Murine myeloid leukemia)	RE (10 µM equivalent of CA) (48–96 h)	Potentiated following effects of VDA: ↑ cell differentiation ↓ cell viability ↓ cell proliferation	↓ ROS ↑ antioxid-ant activity ↑ NADP(H)-quinone reductase	[40]
K-562 (Human leukemia)	6.25–50 µg/mL (48 h)	↓ cell viability IC50 ~12.50 µg/mL		[30]
K-562 (Human leukemia), U937 (Myeloid leukemia)	50 µg/mL (0–96 h)	↓ cell proliferation	↓ AKT1 ↑ Rb2 ↔ ERK2	[42]

VDA (vitamin D analogue), LPS (lipopolysaccharide), NO (nitric oxide), TNFα (tumor necrosis factor α), IL-1β (interleukin 1β), iNOS (inducible nitric oxide synthase), COX2 (cyclooxygenase 2), ROS (reactive oxygen species), NADP (nicotinamide adenine dinucleotide phosphate), Rb2 (retinoblastoma-related gene 2).

**Table 6:** Anticancer effects of Rosemary Extract (RE). In vivo studies.

Animal Model	Dose/Duration	Findings	Mechanism	Reference
SW620 colon xenograft (nude mice)	1 mg/mL in drinking water (32–35 days) ad libitum	↓ tumor size	↓ miR-15b in plasma	[21]
HCT116 colon xenograft (athymic nude mice)	100 mg/kg/day in 100 µL olive oil by oral gavage (4 weeks)	↓ tumor size	↑ Nrf2 expression ↑ sestrin-2 expression	[23]
NMN-induced colon cancer (Sprague-Dawley rats)	1666.6 mg/kg/day (low dose) RE or 3333.3 mg/kg/day (high dose) RE orally (4 months)	Both RE showed comparable effects. Lead to lymphoid cell aggregation in submucosa	↑ cyt C ↑ PCDP4 ↓CEA ↓ CCSA-4 ↓ β-catenin, K-ras, c-myc gene expression	[43]
22RV1 prostate xenograft (athymic nude mice)	100 mg/kg/day in olive oil, orally (22 days)	↓ tumor volume (induces apoptosis)	↓ androgen receptor expression ↓ PSA ↑ CHOP	[33]
DEN-induced liver cancer (F344 rats)	100 mg/kg/day RE intragastrically (5 days) Injected i.p with 20 mg/kg DEN on day 4. Fed normal diet until week 3 (underwent partial hepatectomy)	↑ antioxidant activity	↓ GST positive foci	[44]
Swiss mice exposed to γ-IR (liver)	6Gy γ-IR (once) followed by 1000 mg/kg/day RE orally (5 days)	Delayed onset of IR-induced mortality Attenuated negative IR effects Protective effect on liver and blood	↓ LPx levels ↑ GSH levels	[45]
Myeloid leukemia inoculated mice	1% RE *w/w* in food ad libitum (29 days)	↓ tumor volume ↓ tumor incidence Potentiated VDA ability to ↓ tumor volume		[41]
Myeloid leukemia inoculated mice	4% *w/w* in food ad libitum (15 weeks)	RE alone ↔ median survival time RE+VDA ↑ median survival time	↓ WBC	[40]
DMBA-induced skin cancer (nude mice)	1000 mg/kg/day RE orally in water or by gavage (15 weeks)	↓ tumor number ↓ tumor incidence ↓ tumor burden ↓ tumor yield ↑ latency period	↓ LPx levels ↑ GSH levels	[46]
DMBA-induced skin cancer (nude mice)	500 mg/kg/day RE orally in water or by gavage (15 weeks)	↓ tumor number ↓ tumor diameter ↓ tumor weight	↓ LPx levels ↑ GSH levels	[47]

miR-15b (microRNA 15b), PSA (prostate specific antigen), CHOP (C/EBP homologous protein), VDA (vitamin D analogue), WBC (white blood cell), GST (glutathione *S* transferase), IR (ionizing radiation), LPx (lipid peroxidase), GSH (glutathione), DEN (diethylnitrosamine), DMBA (7,12-dimethylbenz(a)anthracene), NMN (*N*-methylnitrosourea), cyt C (cytochrome C), PCDP4 (programmed cell death protein 4), CEA (carcinoembryonic antigen), CCSA-4 (colon cancer specific antigen 4), LPx (lipid peroxidase), GSH (glutathione).

**Table 7:** Anticancer effects of Carnosic Acid (CA). In vitro studies: colon cancer.

Cell Type	Dose/Duration	Findings	Mechanism	Reference
Caco-2 (Colorectal adenocarcinoma)	1–50 µM CA (48 h)	↓ cell proliferation ↑ cell cycle arrest ↑ cell doubling time IC50 23 µM	↓ cyclin A	[48]
Caco-2 (Colorectal adenocarcinoma), HT-29 (Colorectal adenocarcinoma), LoVo (Colorectal adenocarcinoma)	1–388 µM CA (48 h)	↑ apoptosis ↓ cell adhesion and migration IC50 26.4–92.1 µM (high in Caco2)	↓ MMP-9 and uPA activity, COX-2 expression	[49]
SW480 (Colorectal adenocarcinoma)	25–100 µM CA (6 h)	targets activated β-catenin for proteasomal degradation and destabilizes oncogenic β-catenin	↓ BCL9-β-catenin interaction	[50]
SW620 (Colorectal adenocarcinoma), DLD-1 (Colorectal adenocarcinoma)	2–18 µg/mL (6.02–54.15 µM) CA (48 h)	↓ cell viability	↑ GCNT3. ↓ miR-15b gene expression.	[21]
HT-29 (Colorectal adenocarcinoma)	5–35 µg/mL (15–105 µM) CA (24–72 h)	↓ cell proliferation ↑ cell cycle arrest Alters activity of detoxifying enzymes and metabolites	↑ GSH levels Altered expression of transport and biosynthesis genes ↓ N-acetylputrescine	[51]
HT-29 (Colorectal adenocarcinoma)	1–10 µM CA (24–48 h)	↓ cell viability ↑ cell cycle arrest ↓ triglyceride accumulation of 3T3-L1 adipocytes	↓ p-Akt, cyclin D1, CDK4, Bcl-xL ↑ Bax expression, Ob-R expression	[52]
HT-29 (Colorectal adenocarcinoma), SW480 (Colorectal adenocarcinoma), HGUE-C-1 (Colorectal carcinoma)	30–60 µg/mL (24–48 h) CA fraction of RE (98.7% purity)	↓ cell viability		[22]
HT-29 (Colorectal adenocarcinoma)	12.5 µg/mL (37.6 µM) CA (2–72 h)	↓ cell proliferation ↑ cell cycle arrest ↑ cholesterol accumulation ↑ ROS accumulation	↑ UPR ↑ ER-stress ↓ cell cycle genes Altered cholesterol-modulating genes	[24]
HT-29 (Colorectal adenocarcinoma), HCT116 (Colorectal carcinoma), SW480 (Colorectal adenocarcinoma)	20–100 µM CA (24 h)	↓ cell viability ↑ apoptosis	↑ p53, Bax, casp 3, casp 9, PARP cleavage ↑ ROS generation ↓ MDM2, Bcl-2, Bcl-xL ↓ survivin, cyclins STAT3	[53]
HT-29 (Colorectal adenocarcinoma)	8.3–16.6 µg/mL (25–50 µM) CA (24 h)	↓ cell proliferation	↑ H_2_O_2_ ↑ ROS	[25]

MMP-9 (matrix metallopeptidase 9), uPA (urokinase plasminogen activator), COX-2 (cyclooxygenase 2), BCL9-β (B-cell CLL/lymphoma 9), GCNT3 (glucosaminyl (*N*-Acetyl) transferase 3), GSH (glutathione), CDK4 (cyclin-dependent kinase 4), Bcl-xL (B-cell lymphoma-extra large), Bax (Bcl-2-like protein 4), Ob-R (leptin receptor), ROS (reactive oxygen species), UPR (unfolded protein response), ER (endoplasmic reticulum), casp (caspase), p53 (tumor protein p53), PARP (poly(ADP-ribose) polymerase), MDM2 (mouse double minute 2 homolog), Bcl-2 (B-cell CLL/lymphoma 2), STAT3 (signal transducer and activator of transcription 3), H_2_O_2_ (hydrogen peroxide).

**Table 8:** Anticancer effects of Carnosic Acid (CA). In vitro studies: breast, pancreatic, prostate and ovarian cancer.

Cell Type	Dose/Duration	Findings	Mechanism	Reference
MCF-7 *(ER+)* (Breast adenocarcinoma), MDA-MB-231 (Breast adenocarcinoma)	6.25–50 µg/mL (18.8–150 µM) CA (48 h)	↓ cell viability		[30]
MCF-7 (Breast adenocarcinoma), MDA-MB-468 (Breast adenocarcinoma)	0.5–40 µg/mL (1.5–120 µM) CA (6–96 h)	↓ proliferation ↑ apoptosis ↑ cell cycle arrest IC50: 3µg/mL (9 µM) (88 h)	↑ CYP4F3, GCLC, SLC7A11, CDKN1A expression	[54]
MDA-MB-361 (Breast adenocarcinoma)	20–60 µM CA (24 h)	↑ apoptosis		[55]
MDA-MB-361 (Breast adenocarcinoma)	20 µM CA (24 h)	↓ proliferation ↑ apoptosis	↑ TRAIL-mediated apoptosis ↓ c-FLIP, Bcl-2 ↑ DR5, Bim, PUMA, CHOP	[56]
RINm5F (Insulinoma)	12–100 µg/mL (36.1–300 µM) CA (24–48 h)	↓ cell viability		[27]
MIA-PaCa-2 (Pancreatic carcinoma), PANC-1 (Pancreatic carcinoma)	2–18 µg/mL (6.02–54.15 µM) CA (48 h)	↓ cell viability		[21]
DU145 (Prostate carcinoma), PC3 (Prostate adenocarcinoma)	6.25–50 µg/mL (18.8–150 µM) CA (48h)	↓ cell viability		[30]
PC3 (Prostate adenocarcinoma)	20–100 µM CA (0–72 h)	↓ proliferation ↑ apoptosis	↓ casp 8, casp 9, Bcl-2, Bid, IAP, p-Akt, p-GSK3, NF-κB ↑ casp 3, casp 7, PARP cleavage, Bax, cyt c, PP2A	[58]
LNCaP (Prostate carcinoma), PC3 (Prostate adenocarcinoma), DU-145 (Prostate carcinoma)	10 µM CA (72 h)	↓ proliferation	↓ EpRE/ARE transcription system ↓ PSA secretion	[57]
A2780 (Ovarian carcinoma), A2780CP70 (cisplatin-resistant) (Ovarian carcinoma)	2.5–10 µg/mL (7.2–30 µM) CA (48 h)	↓ cell proliferation Enhanced sensitivity of cisplatin-resistant cells		[34]

CYP4F3 (leukotriene-B(4)omega-hydroxylase 2), GCLC (glutamate-cysteine ligase catalytic subunit), SLC7A11 (solute carrier family 7 member 11), CDKN1A (cyclin-dependent kinase inhibitor 1A), TRAIL (TNF-related apoptosis-inducing ligand), c-FLIP (cellular FLICE (FADD-like-IL-1β-converting enzyme)-inhibiting protein), DR5 (death receptor 5), Bim (Bcl-2-like protein 11), PUMA (p53 upregulated modulator of apoptosis), CHOP (C/EBP homologous protein), casp (caspase), Bcl-2 (B-cell CLL/lymphoma 2), Bid (BH3 interacting-domain), IAP (inhibitor of apoptosis), p-Akt (phosphorylated protein kinase B), p-GSK3 (phosphorylated glycogen synthase kinase 3), NF-κB (nuclear factor kappa B), PARP (poly (ADP-ribose) polymerase), Bax (Bcl-2-like protein 4), cyt c (cytochrome c), PP2A (protein phosphatase 2A), EpRE (electrophile responsive element), ARE (antioxidant response element), PSA (prostate specific antigen).

**Table 9:** Anticancer effects of Carnosic Acid (CA). In vitro studies: liver, lung, skin and kidney cancer.

Cell Type	Dose/Duration	Findings	Mechanism	Reference
Hep-3B (Hepatocellular carcinoma)	6.25–50 µg/mL (18.8–150 µM) CA (48 h)	↓ cell viability		[30]
HepG2 (Hepatocellular carcinoma)	20–100 µM for (12–48 h)	↓ proliferation ↑ apoptosis ↑ autophagic vacuoles and autolysosomes	↑ LC-3 ↓ p-Akt, p-mTOR	[59]
SK-HEP1 (Hepatocellular carcinoma)	20–60 µM CA (24 h)	↑ apoptosis		[55]
SK-HEP1 (Hepatocellular carcinoma)	20 µM CA (24 h)	↓ proliferation ↑ apoptosis	↑ TRAIL-mediated apoptosis ↓ c-FLIP, Bcl-2 ↑ DR5, Bim, PUMA, CHOP	[56]
Rat clone 9 (Normal rat liver)	1–20 µM CA (24 h)	↑ reporter activity of enhancer element GPEI ↑ detoxification systems	↑ GSTP expression ↑ Nrf2 translocation ↑ p38	[60]
Rat clone 9 (Normal rat liver)	1–20 µM CA (0–24 h)	↓ cell survival	↑ NQO1 ↑ Nrf2 ↑ p-p38 ↑ p-ERK	[61]
NCI-H82 (Lung carcinoma; SCLC)	6.25–50 µg/mL (18.8–150 µM) CA (48 h)	↓ cell viability		[30]
HT-1080 (Fibrosarcoma)	25–100 µM CA (4–72 h)	↑ apoptosis ↑ cell cycle arrest ↑ chromatin condensation and DNA fragmentation IC50 9 µM		[62]
BAEC Aortic endothelial cells), HUVEC (Umbilical vein endothelial cells)	25–100 µM CA (4–72 h)	↓ cell survival ↑ apoptosis ↑ cell cycle arrest ↓ migration IC50 36µM	↓ MMP-2 ↓ endothelial cell tubulogenesis.	[62]
B16F10 (Skin melanoma)	2.5–10 µM CA (12 h)	↓ cell migration and adhesion Suppressed mesenchymal markers Induced epithelial markers	↓ MMP-9, TIMP-1, uPA, VCAM-1 ↓ p-Src, p-FAK, p-Akt	[63]
Caki (Kidney clear cell carcinoma)	20–60 µM CA (24 h)	↑ apoptosis Promotes ROS production	↑ PARP cleavage, casp 3, ATF4, CHOP	[55]
Caki (Kidney clear cell carcinoma), AHCN (Kidney renal cell adenocarcinoma), A498 (Kidney carcinoma)	20 µM CA (24 h)	↓ proliferation ↑ apoptosis	↑ TRAIL-mediated apoptosis ↓ c-FLIP, Bcl-2 ↑ DR5, Bim, PUMA, CHOP	[56]

LC3 (light chain 3), p-mTOR (phosphorylated mammalian target of rapamycin), TRAIL (TNF-regulated apoptosis-inducing ligand), c-FLIP (cellular FLICE (FADD-like-IL-1β-converting enzyme)-inhibiting protein), Bcl-2 (B-cell CLL/lymphoma 2), DR5 (death receptor 5), Bim (Bcl-2-like protein 11), PUMA (p53 upregulated modulator of apoptosis), CHOP (C/EBP homologous protein), GSTP (Glutathione *S*-transferase P), Nrf2 (nuclear factor E2-related factor-2), NQO1 (NAD(P)H-quinone oxidoreductase 1), p-ERK (phosphorylated extracellular signal-regulated kinases), MMP-2 (matrix metalloproteinase-2), MMP-9 (matrix metallopeptidase-9), TIMP-1 (TIMP metallopeptidase inhibitor 1), uPA (urokinase plasminogen activator), VCAM-1 (vascular cell adhesion protein 1), p-Src (proto-oncogene tyrosine-protein kinase Src), p-FAK (phosphorylated focal adhesion kinase), PARP (poly(ADP-ribose)polymerase), casp (caspase).

**Table 10:** Anticancer effects of Carnosic Acid (CA). In vitro studies: brain and neural cancer.

Cell Type	Dose/Duration	Findings	Mechanism	Reference
T98G (Glioblastoma)	5–100 µM CA (0–48 h)		↑ NGF synthesis	[64]
T98G (Glioblastoma)	2–50 µM CA (24 h)		↑ NGF synthesis ↑ Nrf2, HO-1, TXNRD1	[65]
IMR-32 (Neuroblastoma)	5–40 µM CA (0–48 h)	↓ cell viability ↑ apoptosis ↑ ROS generation	↑ casp 3, casp 9, PARP, p-p38 ↓ p-ERK	[66]
U373MG (Glioblastoma)	50 µM CA (8 h)	↓ amyloid beta peptide release	↑ α-secretase TACE/ADAM17	[69]
SH-SY5Y (Neuroblastoma)	1 µM CA (12 h)	↑ antioxidant defense ↑ detoxification systems Blocked activation of apoptosis	↑ PI3K/Akt ↓ cytochrome c release ↓ caspase cascade	[67]
SH-SY5Y (Neuroblastoma)	10 µM CA (1 h)	↓ apoptosis	↓ caspase cascade	[68]
GBM (Glioblastoma)	17.5–40 µM CA (48 h)	↓ cell survival ↑ cell cycle arrest ↑ apoptosis	↓ CDK activity ↓ cyclin B1 ↓ RB ↓ SOX2 ↓ GFAP	[70]

NGF (nerve growth factor), Nrf2 (nuclear factor E2-related factor 2), HO-1 (heme oxygenase-1), TXNRD1 (thioredoxin reductase 1), casp (caspase), PARP (poly(ADP-ribose)polymerase), p-ERK (phosphorylated extracellular signal-regulated kinases), TACE (TNF-α converting enzyme), ADAM17 (ADAM metallopeptidase domain 17), PI3K (phosphatidylinositol-4,5-bisphosphate 3-kinase), Akt (protein kinase B), cyt c (cytochrome c), CDK (cyclin dependent kinase), RB (retinoblastoma), SOX2 (sex determining region Y-box 2), GFAP (glial fibrillary acidic protein).

**Table 11:** Anticancer effects of Carnosic Acid (CA). In vitro studies: leukemia.

Cell Type	Dose/Duration	Findings	Mechanism	Reference
HL-60 (Myeloid leukemia)	10 µM CA (0–48 h)	CA potentiated effects of 1,25D ↑ differentiation ↓ proliferation ↑ cell cycle arrest	↑ vitamin D receptor, retinoic acid receptor	[71]
HL-60 (Myeloid leukemia), U937 (Myeloid leukemia)	2.5–10 µM CA (0–48 h)	CA potentiated effects of 1,25D ↑ differentiation ↓ proliferation ↑ cell cycle arrest IC50 6–7µM	↑ p21^Waf1^, p27^Kip1^	[72]
HL-60-G (Myeloid leukemia)	10 µM CA (0–48 h)	CA potentiated effects of 1,25D ↑ differentiation ↓ ROS	↑ GSH ↑ Raf/MAPK/ERK, AP-1	[73]
HL-60 (Myeloid leukemia)	10 µM CA (0–72 h)	CA potentiated effects of 1,25D ↑ differentiation	↑ JNK pathway	[74]
WEHI-3B (Murine myeloid leukemia), HL-60 (Myeloid leukemia), U937 (Myeloid leukemia)	10 µM CA (0–96 h)	CA potentiated effects of 1,25D ↑ differentiation ↓ proliferation ↑ cell cycle arrest		[41]
WEHI-3B D (Murine myeloid leukemia)	10 µM CA (48–96 h)	CA potentiated effects of 1,25D ↑ cell differentiation ↓ cell viability ↓ cell proliferation	↓ ROS ↑ NADP(H)-quinone reductase	[40]
K562 (Myeloid leukemia)	2.5–50 µM CA (24–72 h)	↓ cell viability CA sensitized resistant cells to Adriamycin		[80]
HL-60G (Myeloid leukemia), HL-60-40AF (Myeloid leukemia)	10 µM CA (0–48 h)	CA potentiated effects of 1,25-D ↑ differentiation	↑ JNK1, c-jun-ATF2, C/EBP	[75]
K-562 (Myeloid leukemia)	6.25–50 µg/mL (18.8–150 µM) CA (48 h)	↓ cell viability		[30]
U937 (Myeloid leukemia)	10 µM CA (96 h)	CA potentiated effects of 1,25-D ↑ differentiation	↑ Nrf2, ARE, NADPH,	[77]
HL-60 (Myeloid leukemia), U937 (Myeloid leukemia)	10 µM CA (48 h)	Enhances activity of 1,25D ↑ cell cycle arrest Induces differentiation Sensitizes 1,25D resistant cells	↑ HPK1	[76]
HL-60 (Myeloid leukemia), U937	10 µM CA (48 h)	Enhances activity of doxercalciferol ↑ cell cycle arrest Induces differentiation	↓ microRNA181a	[81]
HL-60 (Myeloid leukemia)	5–25 µM CA (24–72 h)	↓ viability ↑ apoptosis ↑ cell cycle arrest	↑ p27, cleaved casp 9, PTEN expression ↓ p-BAD, p-Akt	[82]
HL-60 (Myeloid leukemia)	25–100 µM CA (4–72 h)	↓ cell survival ↑ apoptosis ↑ cell cycle arrest IC50 5.7 µM	↑ casp 3	[62]
HL-60 (Myeloid leukemia), U937 (Myeloid leukemia), MOLM-13 (Acute monocytic leukemia)	10 µM CA (96 h)	CA potentiated effects of 1,25-D ↑ differentiation		[78]
NB4 (Human promyelocytic leukemia)	5 µM CA (24 h)	Ameliorates arsenic trioxide-induced cytotoxic effects	↑ GSH levels Activation of Nrf2	[79]

1,25-D (1α25-dihydroxyviatminD), GSH (glutathione), Raf (rapidly accelerated fibrosarcoma), MAPK (mitogen-activated protein kinase), ERK (extracellular signal-regulated kinases), AP-1 (activator protein 1), JNK (c-jun N-terminal kinases), ROS (reactive oxygen species), c-jun (v-jun sarcoma virus 17 oncogene), ATF2 (activating transcription factor 2), Nrf2 (nuclear factor E2-regulated factor-2), ARE (antioxidant response element), HPK1 (hematopoietic progenitor kinase 1), casp (caspase), PTEN (phosphatase and tensin homolog).

**Table 12:** Anticancer effects of Carnosic Acid (CA). In vivo studies.

Animal Model	Dose/Duration	Findings	Mechanism	Reference
DMBA-induced oral cancer-hamster	10 mg/kg/day CA (14 weeks)	↓ # of tumors Anti-lipid peroxidative function ↑ detoxification enzymes		[83]
DMBA-induced oral cancer-hamster	10 mg/kg/day CA orally for (14 weeks)	↓ # of tumors	↑ p53, Bax, Bcl-2, casp 3, casp 9 ↓ COX-2, c-fos, NF-κB, cyclin D1	[84]
Human prostate biopsies xenografted into mice	100 mg/mouse dissolved in 100 µL cottonseed oil daily (25 days)	↓ tumor growth		[86]
DMBA-induced oral cancer-hamster	750 µg CA dissolved in 0.1 mL saline (20 µM) daily for (11 weeks)	↓ progression of cancer and development of lesions		[85]
AOM-induced colon cancer-mice	0.01%–0.02% CA fed with a high fat (45%) diet for (11 weeks)	↓ # of tumors ↓ tumor size	↓ insulin, leptin and IGF-1 serum levels compared to mice fed HFD alone ↓ insulin receptor, leptin receptor, p-ERK, cyclin D1, Bcl-xL expression	[52]
K562 leukemia inoculated mouse	1% (*v*/*v*) CA with standard powdered rodent diet *Ad libitum*	↓ # of leukemia cells ↑ apoptotic cells ↑ survival time		[87]

Bax (Bcl-2-like protein 4), Bcl-2 (B-cell CLL/lymphoma 2), casp (caspase), COX2 (cyclooxygenase 2), NF-κB (nuclear factor kappa B), IGF-1 (insulin-like growth factor 1), HFD (high fat diet), p-ERK (phosphorylated extracellular signal-regulated kinase), Bcl-xL (B-cell lymphoma-extra large), # (number).

**Table 13:** Anticancer effects of Rosmarinic Acid (RA). In vitro studies: colon, breast, prostate, ovarian, gastric and skin cancer.

Cell Type	Dose and Duration	Findings	Mechanisms	Reference
HT-29 (Colorectal adenocarcinoma)	5–20 μM RA (1 h)	↓ TPA induced COX2 promoter activity	↓ COX2 protein levels	[88]
HCT15 (Colorectal adenocarcinoma), CO115 (Colorectal carcinoma)	10–100 μM RA (48 h)	↑ apoptosis of HCT15 (50 μM) and CO115 (100 μM)	↓ p-ERK levels in HCT15 cells	[89]
Ls174-T (Colorectal adenocarcinoma)	20–300 μg/mL (55.5–832.6 µM) RA (24 h)	↓ migration rate ↓ adhesion IC50 70 μg/mL	↓ ROS	[90]
CO115 (Colorectal carcinoma)	50 μM RA (24 h)	↓ BCNU-induced DNA damage		[91]
MCF-7 (Breast adenocarcinoma)	60 μM RA (24 h)	↓ cell viability		[92]
MCF7 (Breast adenocarcinoma)	2–200 μM RA (72 h)	↓ DNA methyltransferase activity		[93]
MCF-7 (ER+) (Breast adenocarcinoma), MDA-MB-231 (Breast adenocarcinoma)	6.25–50 µg/mL (17.3–138.8 µM) RA (48 h)	↓ cell viability		[30]
MCF-7/Adr (Breast adenocarcinoma), MCF-7/wt (Breast adenocarcinoma)	0.08–10 mM RA EC values: 0.74 mM (in wt) and 0.81 mM (in Adr resistant)	0.08–0.32 mM RA effective ↑ cytotoxicity to MCF-7 cells		[94]
DU145 (Prostate carcinoma), PC3 (Prostate adenocarcinoma)	6.25–50 µg/mL (17.3–138.8 µM) RA (48h)	↓ cell viability		[30]
A2780 (Ovarian carcinoma), A2780CP70 (Ovarian carcinoma)	2.5–10 µg/mL (6.9–27.8 µM) RA (48 h)	↓ cell proliferation Enhanced sensitivity of cisplatin-resistant cells		[34]
SGC7901/Adr (Gastric carcinoma)	0.096–60 μM RA (48 h)	↓ cell viability Reversed drug resistance	↓ expression of p-glycoprotein ↓ activity of p-glycoprotein	[95]
MKN45 (Gastric carcinoma)	200–300 μM RA	↓ cell viability ↓ Warburg effect	↓ glucose uptake ↓ pro-inflammatory cytokines (IL-6 and STAT3)	[96]
B16 (Skin melanoma)	1–100 μM RA (48 h)	↑ melanin content ↑ tyrosinase expression	↑ phosphorylation of CREB	[97]

TPA (12-*O*-tetradecanoylphorbol-13-acetate), COX2 (cyclooxygenase 2), ERK (extracellular signal-regulated kinases), ROS (reactive oxygen species), BCNU (1,3-*bis*-(2-chloroethyl)-1-nitosourea), IL-6 (interleukin-6), STAT3 (signal transducer and activator of transcription 3) CREB (cAMP response element-binding protein) wt (wild type), Adr (Adriamycin).

**Table 14:** Anticancer effects of Rosmarinic Acid (RA). In vitro studies: liver and lung cancer.

Cell Type	Dose and Duration	Findings	Mechanisms	Reference
HepG2 (Hepatocellular carcinoma)	25–250 μM RA (24 h)	↓ OTA- and AFB-induced cell damage and apoptosis ↓ DNA and protein synthesis inhibition induced by OTA- and AFB-	↓ ROS production ↓ capase-3 activation	[98]
HepG2 (Hepatocellular carcinoma)	5–10 μg/mL (13.9–27.8 µM) RA (72 h)	↑ apoptosis	↑ casp 8, NFBIA, TNFSF9 and Jun mRNA ↓ Bcl-2 mRNA expression	[99]
HepG2 (Hepatocellular carcinoma)	60 μM RA (24 h)	↓ cell viability		[92]
Hep-3B (Hepatocellular carcinoma)	6.25–50 µg/mL (17.3–138.8 µM) RA (48 h)	↓ cell viability		[30]
HepG2 (Hepatocellular carcinoma)	20–80 μM RA (24 h or 4 days)	↔cell viability	↑ translocation of Nrf2 ↑ ARE-luciferin activity ↑ efflux of p-glycoprotein ↑ MRP2 ↑ intracellular ATP	[100]
NCI-H82 (Lung carcinoma; SCLC)	6.25–50 µg/mL (17.3–138.8 µM) RA (48 h)	↓ cell viability		[30]
A549 (Lung adenocarcinoma)	10–500 μM RA (48 h) IC50 198.12	↓ cell proliferation	↓ hCOX2 activity	[101]

OTA (ochratoxin), AFB (Aflatoxin), ROS (reactive oxygen species), casp (caspase), NFBIA (nuclear factor of kappa light polypeptide gene enhancer in B-cells inhibitor-alpha), TNFSF9 (tumor necrosis factor ligand superfamily-member 9), Jun (v-jun sarcoma virus 17 oncogene), Bcl-2 (B-cell CLL/lymphoma 2), Nrf2 (nuclear factor E2-related factor-2), ARE (antioxidant response element), MRP2 (multidrug resistance-associated protein 2), ATP (adenosine triphosphate), hCOX2 (human cyclooxygenase 2).

**Table 15:** Anticancer effects of Rosmarinic Acid (RA). In vitro studies: leukemia.

Cell Type	Dose and Duration	Findings	Mechanisms	Reference
K562 (Myeloid leukemia)	25 μM RA (1 h)	↓ hyperosmotic-mediated ROS/RNS production and apoptosis		[102]
U937 (Myeloid leukemia)	60 μM RA (24 h)	↑ TNF-α induced apoptosis	↓ NF-κB activation ↓ ROS production ↑ caspases	[92]
K562 (Myeloid leukemia)	6.25–50 µg/mL (17.3–138.8 µM) (48 h)	↓ cell viability		[30]
K562 (Myeloid leukemia), U937 (Myeloid leukemia)	0.2 mM RA (48 h)	Not tested on proliferation	↔ AKT1 ↔ ERK2	[42]
NB4 (Human promyelocytic leukemia)	40 μM RA (72 h)	↑ ATRA-induced macrophage differentiation	↑ expression of CD11b	[103]
HL-60 (Myeloid leukemia)	50–150 μM RA (24–72 h)	↓ cell growth ↑ apoptosis IC50 147 μM (24 h), 74 μM (48 h), 69 μM (72 h)	↓ dNTP levels	[104]
CCRF-CEM (Lymphoblastic leukemia), CEM/ADR5000 (Lymphoblastic leukemia)	3–100 μM RA (72 h)	↑ cytotoxicity ↑ apoptosis and necrosis ↑ cell cycle arrest ↑ caspase-independent apoptosis	↑ PARP-cleavage Blocked p65 nuclear translocation from the cytosol	[105]
HL-60 (Myeloid leukemia)	0.07–2.2 mM RA (72 h)	DNA protection and anticarcinogenic effects		[106]

ROS (reactive oxygen species), RNS (reactive nitrogen species), TNF-α (tumor necrosis factor-alpha), NF-κB (nuclear factor kappa-light-chain-enhancer of activated B cells), Akt (protein kinase B), ERK (extracellular signal-regulated kinases), ATRA (all-*trans* retinoic acid), dNTP (deoxy-nucleoside triphosphate), PARP (poly(ADP-ribose) polymerase).

**Table 16:** Anticancer effects of Rosmarinic Acid (RA). In vivo studies.

Animal Model	Dose and Duration	Findings	Mechanisms	Reference
Seven-Nine week old male Balb/c mice	0.25, 0.5, 1.0 and 1.35 mg/mouse (30 months) before TPA treatment	↓ myeloperoxidase activity	↓ COX2 induction	[107]
C57BL/6 mice implanted with Lewis lung carcinoma	1, 2 and 4 mg/kg RA (20 days)	↓ tumor growth		[90]
Golden Syrian hamsters	100 mg/kg RA (14 weeks)	Completely prevented tumor formation in DMBA-treated hamsters	↓ p53 ↓ Bcl-2	[108]
C57BL/6J Min/+ (Apc^Min^) mice	360 mg/kg RA (8 weeks)	↓ the frequency of large adenomas	↑ levels of parent compound in plasma	[109]
DMH induced colon cancer (Albino Wistar male rats)	2.5–10 mg/kg RA (16 weeks) through intragastric intubation	↓ DMH induced aberrant crypt foci	↓ DMH induced increase in bacterial enzymes	[110]
DMBA induced skin cancer (Swiss albino mice)	100 mg/kg RA administered (1 weeks) before DMBA treatment	↓ skin tumors	↑ status of phase I (cyt p450) detoxification agents ↑ status of phase II (GST, GR, GSH) detoxification agents. Restored activity levels of casp 3, casp 9, p53 and Bcl-2.	[111]
DMH induced colon cancer (Male Wistar rats)	2.5, 5 and 10 mg/kg RA (4 weeks)	↓ DMH induced aberrant crypt foci, number of polyps, reversed the markers of oxidative stress, antioxidant status, CYP450 content and PNPH activity		[112]
Five month old Syrian hamsters	1.3 mg/mL RA (2 weeks) pretreatment	↓ incidence of tumors ↑ differentiation ↓ scores in the tumor invasion front grading system.		[113]
5 week old male nude Balb/c mice incubated sub-cutaneously with MKN45 cells into their flanks.	2 mg/kg RA via celiac injection daily (14 days)	↓ Warburg effect	↓ glucose uptake	[114]
DMH induced colon cancer (Male Wistar rats)	5 mg/kg RA orally (30 weeks)	↓ DMH induced colon tumor formation	↓ TNF-α ↓ IL-6 ↓ COX2	[96]

TPA (12-*O*-tetradecanoylpheorbol-13-acetate), COX2 (cyclooxygenase 2), DMBA (7,12-dimethylbenz(a)anthracene), DMH (1,2-dimethylhydrazine), p53 (tumor protein p53), casp (caspase), Bcl-2 (B-cell CLL/lymphoma 2), CYP450 (cytochrome p450), GST (Glutathione *S*-transferase), GR (glucocorticoid receptor), GSH (glutathione), PNPH (p-nitrophenol hydroxylase), TNF-α (tumor necrosis factor alpha), IL-6 (interleukin-6).

## References

[1] Hanahan D., Weinberg R.A. (2011). Hallmarks of Cancer: The Next Generation. Cell.

[2] Bhowmick N.A., Neilson E.G., Moses H.L. (2004). Stromal fibroblasts in cancer initiation and progression. Nature.

[3] Cheng N., Chytil A., Shyr Y., Joly A., Moses H.L. (2008). Transforming Growth Factor-β Signaling-Deficient Fibroblasts Enhance Hepatocyte Growth Factor Signaling in Mammary Carcinoma Cells to Promote Scattering and Invasion. Mol. Cancer Res..

[4] Da Rocha A.B., Lopes R.M., Schwartsmann G. (2001). Natural products in anticancer therapy. Curr. Opin. Pharmacol..

[5] Storozhuk Y., Hopmans S.N., Sanli T., Barron C., Tsiani E., Cutz J.-C., Pond G., Wright J., Singh G., Tsakiridis T. (2013). Metformin inhibits growth and enhances radiation response of non-small cell lung cancer (NSCLC) through ATM and AMPK. Br. J. Cancer.

[6] Bai Y., Mao Q.-Q., Qin J., Zheng X.-Y., Wang Y.-B., Yang K., Shen H.-F., Xie L.-P. (2010). Resveratrol induces apoptosis and cell cycle arrest of human T24 bladder cancer cells in vitro and inhibits tumor growth in vivo. Cancer Sci..

[7] Rashid A., Liu C., Sanli T., Tsiani E., Singh G., Bristow R.G., Dayes I., Lukka H., Wright J., Tsakiridis T. (2011). Resveratrol enhances prostate cancer cell response to ionizing radiation. Modulation of the AMPK, Akt and mTOR pathways. Radiat. Oncol..

[8] Varoni E.M., Lo Faro A.F., Sharifi-Rad J., Iriti M. (2016). Anticancer Molecular Mechanisms of Resveratrol. Front. Nutr..

[9] Aggarwal B.B., Bhardwaj A., Aggarwal R.S., Seeram N.P., Shishodia S., Takada Y. (2004). Role of Resveratrol in Prevention and Therapy of Cancer: Preclinical and Clinical Studies. Anticancer Res..

[10] Barron C.C., Moore J., Tsakiridis T., Pickering G., Tsiani E. (2014). Inhibition of human lung cancer cell proliferation and survival by wine. Cancer Cell Int..

[11] Cuvelier M.E., Berset C., Richard H. (1994). Antioxidant Constituents in Sage (*Salvia officinalis*). J. Agric. Food Chem..

[12] González-Vallinas M., Reglero G., Ramírez de Molina A. (2015). Rosemary (*Rosmarinus officinalis* L.) Extract as a Potential Complementary Agent in Anticancer Therapy. Nutr. Cancer.

[13] Petiwala S.M., Puthenveetil A.G., Johnson J.J. (2013). Polyphenols from the Mediterranean herb rosemary (*Rosmarinus officinalis*) for prostate cancer. Front. Pharmacol..

[14] Petiwala S.M., Johnson J.J. (2015). Diterpenes from rosemary (*Rosmarinus officinalis*): Defining their potential for anti-cancer activity. Cancer Lett..

[15] Slamenova D., Kuboskova K., Horvathova E., Robichova S. (2002). Rosemary-stimulated reduction of DNA strand breaks and FPG-sensitive sites in mammalian cells treated with H_2_O_2_ or visible light-excited Methylene Blue. Cancer Lett..

[16] Yi W., Wetzstein H.Y. (2011). Anti-tumorigenic activity of five culinary and medicinal herbs grown under greenhouse conditions and their combination effects. J. Sci. Food Agric..

[17] Ibáñez C., Simó C., García-Cañas V., Gómez-Martínez Á., Ferragut J.A., Cifuentes A. (2012). CE/LC-MS multiplatform for broad metabolomic analysis of dietary polyphenols effect on colon cancer cells proliferation. Electrophoresis.

[18] Đilas S., Knez Ž., Četojević-Simin D., Tumbas V., Škerget M., Čanadanović-Brunet J., Ćetković G. (2012). In vitro antioxidant and antiproliferative activity of three rosemary (*Rosmarinus officinalis* L.) extract formulations. Int. J. Food Sci. Technol..

[19] Valdés A., Garcia-Canas V., Rocamora-Reverte L., Gomez-Martinez A., Ferragut J.A., Cifuentes A. (2013). Effect of rosemary polyphenols on human colon cancer cells: Transcriptomic profiling and functional enrichment analysis. Genes Nutr..

[20] González-Vallinas M., Molina S., Vicente G., de la Cueva A., Vargas T., Santoyo S., García-Risco M.R., Fornari T., Reglero G., Ramírez de Molina A. (2013). Antitumor effect of 5-fluorouracil is enhanced by rosemary extract in both drug sensitive and resistant colon cancer cells. Pharmacol. Res..

[21] González-Vallinas M., Molina S., Vicente G., Zarza V., Martín-Hernández R., García-Risco M.R., Fornari T., Reglero G., de Molina A.R. (2014). Expression of MicroRNA-15b and the Glycosyltransferase GCNT3 Correlates with Antitumor Efficacy of Rosemary Diterpenes in Colon and Pancreatic Cancer. PLoS ONE.

[22] Borrás-Linares I., Pérez-Sánchez A., Lozano-Sánchez J., Barrajón-Catalán E., Arráez-Román D., Cifuentes A., Micol V., Carretero A.S. (2015). A bioguided identification of the active compounds that contribute to the antiproliferative/cytotoxic effects of rosemary extract on colon cancer cells. Food Chem. Toxicol. Int. J. Publ. Br. Ind. Biol. Res. Assoc..

[23] Yan M., Li G., Petiwala S.M., Householter E., Johnson J.J. (2015). Standardized rosemary (*Rosmarinus officinalis*) extract induces Nrf2/sestrin-2 pathway in colon cancer cells. J. Funct. Foods.

[24] Valdés A., Sullini G., Ibáñez E., Cifuentes A., García-Cañas V. (2015). Rosemary polyphenols induce unfolded protein response and changes in cholesterol metabolism in colon cancer cells. J. Funct. Foods.

[25] Valdés A., García-Cañas V., Koçak E., Simó C., Cifuentes A. (2016). Foodomics study on the effects of extracellular production of hydrogen peroxide by rosemary polyphenols on the anti-proliferative activity of rosemary polyphenols against HT-29 cells. Electrophoresis.

[26] Valdés A., Artemenko K.A., Bergquist J., García-Cañas V., Cifuentes A. (2016). Comprehensive Proteomic Study of the Antiproliferative Activity of a Polyphenol-Enriched Rosemary Extract on Colon Cancer Cells Using Nanoliquid Chromatography-Orbitrap MS/MS. J. Proteome Res..

[27] Kontogianni V.G., Tomic G., Nikolic I., Nerantzaki A.A., Sayyad N., Stosic-Grujicic S., Stojanovic I., Gerothanassis I.P., Tzakos A.G. (2013). Phytochemical profile of *Rosmarinus officinalis* and *Salvia officinalis* extracts and correlation to their antioxidant and anti-proliferative activity. Food Chem..

[28] Alexandrov K., Rojas M., Rolando C. (2006). DNA damage by benzo(a)pyrene in human cells is increased by cigarette smoke and decreased by a filter containing rosemary extract, which lowers free radicals. Cancer Res..

[29] Cheung S., Tai J. (2007). Anti-proliferative and antioxidant properties of rosemary *Rosmarinus officinalis*. Oncol. Rep..

[30] Yesil-Celiktas O., Sevimli C., Bedir E., Vardar-Sukan F. (2010). Inhibitory Effects of Rosemary Extracts, Carnosic Acid and Rosmarinic Acid on the Growth of Various Human Cancer Cell Lines. Plant Foods Hum. Nutr..

[31] Mothana R.A.A., Kriegisch S., Harms M., Wende K., Lindequist U. (2011). Assessment of selected Yemeni medicinal plants for their in vitro antimicrobial, anticancer, and antioxidant activities. Pharm. Biol..

[32] González-Vallinas M., Molina S., Vicente G., Sánchez-Martínez R., Vargas T., García-Risco M.R., Fornari T., Reglero G., Ramírez de Molina A. (2014). Modulation of estrogen and epidermal growth factor receptors by rosemary extract in breast cancer cells. Electrophoresis.

[33] Petiwala S.M., Berhe S., Li G., Puthenveetil A.G., Rahman O., Nonn L., Johnson J.J. (2014). Rosemary (*Rosmarinus officinalis*) Extract Modulates CHOP/GADD153 to Promote Androgen Receptor Degradation and Decreases Xenograft Tumor Growth. PLoS ONE.

[34] Tai J., Cheung S., Wu M., Hasman D. (2012). Antiproliferation effect of Rosemary (*Rosmarinus officinalis*) on human ovarian cancer cells in vitro. Phytomedicine.

[35] Berrington D., Lall N. (2012). Anticancer Activity of Certain Herbs and Spices on the Cervical Epithelial Carcinoma (HeLa) Cell Line. Evid. Based Complement. Alternat. Med..

[36] Wang W., Li N., Luo M., Zu Y., Efferth T. (2012). Antibacterial Activity and Anticancer Activity of *Rosmarinus officinalis* L. Essential Oil Compared to That of Its Main Components. Molecules.

[37] Peng C.-H., Su J.-D., Chyau C.-C., Sung T.-Y., Ho S.-S., Peng C.-C., Peng R.Y. (2007). Supercritical Fluid Extracts of Rosemary Leaves Exhibit Potent Anti-Inflammation and Anti-Tumor Effects. Biosci. Biotechnol. Biochem..

[38] Vicente G., Molina S., González-Vallinas M., García-Risco M.R., Fornari T., Reglero G., de Molina A.R. (2013). Supercritical rosemary extracts, their antioxidant activity and effect on hepatic tumor progression. J. Supercrit. Fluids.

[39] Moore J., Megaly M., MacNeil A.J., Klentrou P., Tsiani E. (2016). Rosemary extract reduces Akt/mTOR/p70S6K activation and inhibits proliferation and survival of A549 human lung cancer cells. Biomed. Pharmacother..

[40] Shabtay A., Sharabani H., Barvish Z., Kafka M., Amichay D., Levy J., Sharoni Y., Uskokovic M.R., Studzinski G.P., Danilenko M. (2008). Synergistic Antileukemic Activity of Carnosic Acid-Rich Rosemary Extract and the 19-nor Gemini Vitamin D Analogue in a Mouse Model of Systemic Acute Myeloid Leukemia. Oncology.

[41] Sharabani H., Izumchenko E., Wang Q., Kreinin R., Steiner M., Barvish Z., Kafka M., Sharoni Y., Levy J., Uskokovic M. (2006). Cooperative antitumor effects of vitamin D3 derivatives and rosemary preparations in a mouse model of myeloid leukemia. Int. J. Cancer.

[42] Okumura N., Yoshida H., Nishimura Y., Kitagishi Y., Matsuda S. (2012). Terpinolene, a component of herbal sage, downregulates AKT1 expression in K562 cells. Oncol. Lett..

[43] Ahmad H.H., Hamza A.H., Hassan A.Z., Sayed A.H. (2013). Promising therapeutic role of *Rosmarinus officinalis* successive methanolic fraction against colorectal cancer. Int. J. Pharm. Pharm. Sci..

[44] Kitano M., Wanibuchi H., Kikuzaki H., Nakatani N., Imaoka S., Funae Y., Hayashi S., Fukushima S. (2000). Chemopreventive effects of coumaperine from pepper on the initiation stage of chemical hepatocarcinogenesis in the rat. Jpn. J. Cancer Res. Gann.

[45] Soyal D., Jindal A., Singh I., Goyal P.K. (2007). Modulation of radiation-induced biochemical alterations in mice by rosemary (*Rosemarinus officinalis*) extract. Phytomed. Int. J. Phytother. Phytopharm..

[46] Sancheti G., Goyal P. (2006). Modulatory influence of *Rosemarinus officinalis* on DMBA-induced mouse skin tumorigenesis. Asian Pac. J. Cancer Prev..

[47] Sancheti G., Goyal P.K. (2006). Effect of *Rosmarinus officinalis* in modulating 7,12-dimethylbenz(a)anthracene induced skin tumorigenesis in mice. Phytother. Res..

[48] Visanji J.M., Thompson D.G., Padfield P.J. (2006). Induction of G2/M phase cell cycle arrest by carnosol and carnosic acid is associated with alteration of cyclin A and cyclin B1 levels. Cancer Lett..

[49] Barni M.V., Carlini M.J., Cafferata E.G., Puricelli L., Moreno S. (2012). Carnosic acid inhibits the proliferation and migration capacity of human colorectal cancer cells. Oncol. Rep..

[50] De la Roche M., Rutherford T.J., Gupta D., Veprintsev D.B., Saxty B., Freund S.M., Bienz M. (2012). An intrinsically labile α-helix abutting the BCL9-binding site of β-catenin is required for its inhibition by carnosic acid. Nat. Commun..

[51] Valdés A., García-Cañas V., Simó C., Ibáñez C., Micol V., Ferragut J.A., Cifuentes A. (2014). Comprehensive Foodomics Study on the Mechanisms Operating at Various Molecular Levels in Cancer Cells in Response to Individual Rosemary Polyphenols. Anal. Chem..

[52] Kim Y.-J., Kim J.-S., Seo Y.-R., Park J.-H.Y., Choi M.-S., Sung M.-K. (2014). Carnosic acid suppresses colon tumor formation in association with anti-adipogenic activity. Mol. Nutr. Food Res..

[53] Kim D.-H., Park K.-W., Chae I.G., Kundu J., Kim E.-H., Kundu J.K., Chun K.-S. (2016). Carnosic acid inhibits STAT3 signaling and induces apoptosis through generation of ROS in human colon cancer HCT116 cells. Mol. Carcinog..

[54] Einbond L.S., Wu H., Kashiwazaki R., He K., Roller M., Su T., Wang X., Goldsberry S. (2012). Carnosic acid inhibits the growth of ER-negative human breast cancer cells and synergizes with curcumin. Fitoterapia.

[55] Min K.-J., Jung K.-J., Kwon T.K. (2014). Carnosic Acid Induces Apoptosis Through Reactive Oxygen Species-mediated Endoplasmic Reticulum Stress Induction in Human Renal Carcinoma Caki Cells. J. Cancer Prev..

[56] Jung K.-J., Min K., Bae J.H., Kwon T.K. (2015). Carnosic acid sensitized TRAIL-mediated apoptosis through down-regulation of c-FLIP and Bcl-2 expression at the post translational levels and CHOP-dependent up-regulation of DR5, Bim, and PUMA expression in human carcinoma caki cells. Oncotarget.

[57] Linnewiel-Hermoni K., Khanin M., Danilenko M., Zango G., Amosi Y., Levy J., Sharoni Y. (2015). The anti-cancer effects of carotenoids and other phytonutrients resides in their combined activity. Arch. Biochem. Biophys..

[58] Kar S., Palit S., Ball W.B., Das P.K. (2012). Carnosic acid modulates Akt/IKK/NF-κB signaling by PP2A and induces intrinsic and extrinsic pathway mediated apoptosis in human prostate carcinoma PC-3 cells. Apoptosis Int. J. Program. Cell Death.

[59] Gao Q., Liu H., Yao Y., Geng L., Zhang X., Jiang L., Shi B., Yang F. (2014). Carnosic acid induces autophagic cell death through inhibition of the Akt/mTOR pathway in human hepatoma cells. J. Appl. Toxicol..

[60] Lin C.-Y., Wu C.-R., Chang S.-W., Wang Y.-J., Wu J.-J., Tsai C.-W. (2015). Induction of the pi class of glutathione S-transferase by carnosic acid in rat Clone 9 cells via the p38/Nrf2 pathway. Food Funct..

[61] Tsai C.-W., Lin C.-Y., Wang Y.-J. (2011). Carnosic acid induces the NAD(P)H: Quinone oxidoreductase 1 expression in rat clone 9 cells through the p38/nuclear factor erythroid-2 related factor 2 pathway. J. Nutr..

[62] López-Jiménez A., García-Caballero M., Medina M.Á., Quesada A.R. (2013). Anti-angiogenic properties of carnosol and carnosic acid, two major dietary compounds from rosemary. Eur. J. Nutr..

[63] Park S.Y., Song H., Sung M.-K., Kang Y.-H., Lee K.W., Park J.H.Y. (2014). Carnosic Acid Inhibits the Epithelial-Mesenchymal Transition in B16F10 Melanoma Cells: A Possible Mechanism for the Inhibition of Cell Migration. Int. J. Mol. Sci..

[64] Kosaka K., Yokoi T. (2003). Carnosic acid, a component of rosemary (*Rosmarinus officinalis* L.), promotes synthesis of nerve growth factor in T98G human glioblastoma cells. Biol. Pharm. Bull..

[65] Mimura J., Kosaka K., Maruyama A., Satoh T., Harada N., Yoshida H., Satoh K., Yamamoto M., Itoh K. (2011). Nrf2 regulates NGF mRNA induction by carnosic acid in T98G glioblastoma cells and normal human astrocytes. J. Biochem..

[66] Tsai C.-W., Lin C.-Y., Lin H.-H., Chen J.-H. (2011). Carnosic acid, a rosemary phenolic compound, induces apoptosis through reactive oxygen species-mediated p38 activation in human neuroblastoma IMR-32 cells. Neurochem. Res..

[67] De Oliveira M.R., Ferreira G.C., Schuck P.F., dal Bosco S.M. (2015). Role for the PI3K/Akt/Nrf2 signaling pathway in the protective effects of carnosic acid against methylglyoxal-induced neurotoxicity in SH-SY5Y neuroblastoma cells. Chem. Biol. Interact..

[68] Meng P., Yoshida H., Tanji K., Matsumiya T., Xing F., Hayakari R., Wang L., Tsuruga K., Tanaka H., Mimura J. (2015). Carnosic acid attenuates apoptosis induced by amyloid-β 1–42 or 1–43 in SH-SY5Y human neuroblastoma cells. Neurosci. Res..

[69] Yoshida H., Meng P., Matsumiya T., Tanji K., Hayakari R., Xing F., Wang L., Tsuruga K., Tanaka H., Mimura J. (2014). Carnosic acid suppresses the production of amyloid-β 1–42 and 1–43 by inducing an α-secretase TACE/ADAM17 in U373MG human astrocytoma cells. Neurosci. Res..

[70] Cortese K., Daga A., Monticone M., Tavella S., Stefanelli A., Aiello C., Bisio A., Bellese G., Castagnola P. (2016). Carnosic acid induces proteasomal degradation of Cyclin B1, RB and SOX2 along with cell growth arrest and apoptosis in GBM cells. Phytomed. Int. J. Phytother. Phytopharm..

[71] Danilenko M., Wang X., Studzinski G.P. (2001). Carnosic acid and promotion of monocytic differentiation of HL60-G cells initiated by other agents. J. Natl. Cancer Inst..

[72] Steiner M. (2001). Carnosic Acid Inhibits Proliferation and Augments Differentiation of Human Leukemic Cells Induced by 1,25-Dihydroxyvitamin Dsub3 and Retinoic Acid. Nutr. Cancer.

[73] Danilenko M., Wang Q., Wang X., Levy J., Sharoni Y., Studzinski G.P. (2003). Carnosic acid potentiates the antioxidant and prodifferentiation effects of 1alpha,25-dihydroxyvitamin D3 in leukemia cells but does not promote elevation of basal levels of intracellular calcium. Cancer Res..

[74] Wang Q., Harrison J.S., Uskokovic M., Kutner A., Studzinski G.P. (2005). Translational study of vitamin D differentiation therapy of myeloid leukemia: Effects of the combination with a p38 MAPK inhibitor and an antioxidant. Leukemia.

[75] Chen-Deutsch X., Garay E., Zhang J., Harrison J.S., Studzinski G.P. (2009). c-Jun *N*-terminal kinase 2 (JNK2) antagonizes the signaling of differentiation by JNK1 in human myeloid leukemia cells resistant to vitamin D. Leuk. Res..

[76] Chen-Deutsch X., Studzinski G.P. (2012). Dual role of hematopoietic progenitor kinase 1 (HPK1) as a positive regulator of 1α,25-dihydroxyvitamin D-induced differentiation and cell cycle arrest of AML cells and as a mediator of vitamin D resistance. Cell. Cycle Georget. Tex.

[77] Bobilev I., Novik V., Levi I., Shpilberg O., Levy J., Sharoni Y., Studzinski G.P., Danilenko M. (2011). The Nrf2 transcription factor is a positive regulator of myeloid differentiation of acute myeloid leukemia cells. Cancer Biol. Ther..

[78] Nachliely M., Sharony E., Kutner A., Danilenko M. (2016). Novel analogs of 1,25-dihydroxyvitamin D2 combined with a plant polyphenol as highly efficient inducers of differentiation in human acute myeloid leukemia cells. J. Steroid Biochem. Mol. Biol..

[79] Nishimoto S., Suzuki T., Koike S., Yuan B., Takagi N., Ogasawara Y. (2016). Nrf2 activation ameliorates cytotoxic effects of arsenic trioxide in acute promyelocytic leukemia cells through increased glutathione levels and arsenic efflux from cells. Toxicol. Appl. Pharmacol..

[80] Yu X.-N., Chen X.-L., Li H., Li X.-X., Li H.-Q., Jin W.-R. (2008). Reversion of P-glycoprotein-mediated multidrug resistance in human leukemic cell line by carnosic acid. Chin. J. Physiol..

[81] Duggal J., Harrison J.S., Studzinski G.P., Wang X. (2012). Involvement of microRNA181a in differentiation and cell cycle arrest induced by a plant-derived antioxidant carnosic acid and vitamin D analog doxercalciferol in human leukemia cells. MicroRNA Shāriqah. United Arab Emir..

[82] Wang R., Cong W., Guo G., Li X., Chen X., Yu X., Li H. (2012). Synergism between carnosic acid and arsenic trioxide on induction of acute myeloid leukemia cell apoptosis is associated with modulation of PTEN/Akt signaling pathway. Chin. J. Integr. Med..

[83] Manoharan S., Vasanthaselvan M., Silvan S., Baskaran N., Kumar Singh A., Vinoth Kumar V. (2010). Carnosic acid: A potent chemopreventive agent against oral carcinogenesis. Chem. Biol. Interact..

[84] Rajasekaran D., Manoharan S., Silvan S., Vasudevan K., Baskaran N., Palanimuthu D. (2012). Proapoptotic, anti-cell proliferative, anti-inflammatory and anti-angiogenic potential of carnosic acid during 7,12 dimethylbenz[a]anthracene-induced hamster buccal pouch carcinogenesis. Afr. J. Tradit. Complement. Altern. Med. AJTCAM Afr. Netw. Ethnomed..

[85] Gómez-García F., López-Jornet M., Álvarez-Sánchez N., Castillo-Sánchez J., Benavente-García O., Vicente Ortega V. (2013). Effect of the phenolic compounds apigenin and carnosic acid on oral carcinogenesis in hamster induced by DMBA. Oral Dis..

[86] Petiwala S.M., Li G., Bosland M.C., Lantvit D.D., Petukhov P.A., Johnson J.J. (2016). Carnosic acid promotes degradation of the androgen receptor and is regulated by the unfolded protein response pathway in vitro and in vivo. Carcinogenesis.

[87] Wang L.-Q., Wang R., Li X.-X., Yu X.-N., Chen X.-L., Li H. (2015). The anti-leukemic effect of carnosic acid combined with Adriamycin in a K562/A02/SCID leukemia mouse model. Int. J. Clin. Exp. Med..

[88] Scheckel K.A., Degner S.C., Romagnolo D.F. (2008). Rosmarinic acid antagonizes activator protein-1-dependent activation of cyclooxygenase-2 expression in human cancer and nonmalignant cell lines. J. Nutr..

[89] Xavier C.P.R., Lima C.F., Fernandes-Ferreira M., Pereira-Wilson C. (2009). *Salvia fruticosa*, *Salvia officinalis*, and rosmarinic acid induce apoptosis and inhibit proliferation of human colorectal cell lines: The role in MAPK/ERK pathway. Nutr. Cancer.

[90] Xu Y., Xu G., Liu L., Xu D., Liu J. (2010). Anti-invasion effect of rosmarinic acid via the extracellular signal-regulated kinase and oxidation-reduction pathway in Ls174-T cells. J. Cell. Biochem..

[91] Ramos A.A., Pedro D., Collins A.R., Pereira-Wilson C. (2012). Protection by Salvia extracts against oxidative and alkylation damage to DNA in human HCT15 and CO115 cells. J. Toxicol. Environ. Health A.

[92] Moon D.-O., Kim M.-O., Lee J.-D., Choi Y.H., Kim G.-Y. (2010). Rosmarinic acid sensitizes cell death through suppression of TNF-α-induced NF-κB activation and ROS generation in human leukemia U937 cells. Cancer Lett..

[93] Paluszczak J., Krajka-Kuźniak V., Baer-Dubowska W. (2010). The effect of dietary polyphenols on the epigenetic regulation of gene expression in MCF7 breast cancer cells. Toxicol. Lett..

[94] Berdowska I., Zieliński B., Fecka I., Kulbacka J., Saczko J., Gamian A. (2013). Cytotoxic impact of phenolics from Lamiaceae species on human breast cancer cells. Food Chem..

[95] Li F.-R., Fu Y.-Y., Jiang D.-H., Wu Z., Zhou Y.-J., Guo L., Dong Z.-M., Wang Z.-Z. (2013). Reversal effect of rosmarinic acid on multidrug resistance in SGC7901/Adr cell. J. Asian Nat. Prod. Res..

[96] Han S., Yang S., Cai Z., Pan D., Li Z., Huang Z., Zhang P., Zhu H., Lei L., Wang W. (2015). Anti-Warburg effect of rosmarinic acid via miR-155 in gastric cancer cells. Drug Des. Dev. Ther..

[97] Lee J., Kim Y.S., Park D. (2007). Rosmarinic acid induces melanogenesis through protein kinase A activation signaling. Biochem. Pharmacol..

[98] Renzulli C., Galvano F., Pierdomenico L., Speroni E., Guerra M.C. (2004). Effects of rosmarinic acid against aflatoxin B1 and ochratoxin-A-induced cell damage in a human hepatoma cell line (HepG2). J. Appl. Toxicol..

[99] Lin C.-S., Kuo C.-L., Wang J.-P., Cheng J.-S., Huang Z.-W., Chen C.-F. (2007). Growth inhibitory and apoptosis inducing effect of *Perilla frutescens* extract on human hepatoma HepG2 cells. J. Ethnopharmacol..

[100] Wu J., Zhu Y., Li F., Zhang G., Shi J., Ou R., Tong Y., Liu Y., Liu L., Lu L. (2016). Spica prunellae and its marker compound rosmarinic acid induced the expression of efflux transporters through activation of Nrf2-mediated signaling pathway in HepG2 cells. J. Ethnopharmacol..

[101] Tao L., Wang S., Zhao Y., Sheng X., Wang A., Zheng S., Lu Y. (2014). Phenolcarboxylic acids from medicinal herbs exert anticancer effects through disruption of COX-2 activity. Phytomed. Int. J. Phytother. Phytopharm..

[102] Aquilano K., Filomeni G., Di Renzo L., Vito M.D., Stefano C.D., Salimei P.S., Ciriolo M.R., Marfè G. (2007). Reactive oxygen and nitrogen species are involved in sorbitol-induced apoptosis of human erithroleukaemia cells K562. Free Radic. Res..

[103] Heo S.-K., Noh E.-K., Yoon D.-J., Jo J.-C., Koh S., Baek J.H., Park J.-H., Min Y.J., Kim H. (2015). Rosmarinic acid potentiates ATRA-induced macrophage differentiation in acute promyelocytic leukemia NB4 cells. Eur. J. Pharmacol..

[104] Saiko P., Steinmann M.-T., Schuster H., Graser G., Bressler S., Giessrigl B., Lackner A., Grusch M., Krupitza G., Bago-Horvath Z. (2015). Epigallocatechin gallate, ellagic acid, and rosmarinic acid perturb dNTP pools and inhibit de novo DNA synthesis and proliferation of human HL-60 promyelocytic leukemia cells: Synergism with arabinofuranosylcytosine. Phytomedicine.

[105] Wu C.-F., Hong C., Klauck S.M., Lin Y.-L., Efferth T. (2015). Molecular mechanisms of rosmarinic acid from *Salvia miltiorrhiza* in acute lymphoblastic leukemia cells. J. Ethnopharmacol..

[106] Lozano-Baena M.-D., Tasset I., Muñoz-Serrano A., Alonso-Moraga Á., de Haro-Bailón A. (2016). Cancer Prevention and Health Benefices of Traditionally Consumed *Borago officinalis* Plants. Nutrients.

[107] Osakabe N., Yasuda A., Natsume M., Yoshikawa T. (2004). Rosmarinic acid inhibits epidermal inflammatory responses: Anticarcinogenic effect of *Perilla frutescens* extract in the murine two-stage skin model. Carcinogenesis.

[108] Anusuya C., Manoharan S. (2011). Antitumor initiating potential of rosmarinic acid in 7,12-dimethylbenz(a)anthracene-induced hamster buccal pouch carcinogenesis. J. Environ. Pathol. Toxicol. Oncol. Off. Organ Int. Soc. Environ. Toxicol. Cancer.

[109] Karmokar A., Marczylo T.H., Cai H., Steward W.P., Gescher A.J., Brown K. (2012). Dietary intake of rosmarinic acid by Apc(Min) mice, a model of colorectal carcinogenesis: Levels of parent agent in the target tissue and effect on adenoma development. Mol. Nutr. Food Res..

[110] Karthikkumar V., Sivagami G., Vinothkumar R., Rajkumar D., Nalini N. (2012). Modulatory efficacy of rosmarinic acid on premalignant lesions and antioxidant status in 1,2-dimethylhydrazine induced rat colon carcinogenesis. Environ. Toxicol. Pharmacol..

[111] Sharmila R., Manoharan S. (2012). Anti-tumor activity of rosmarinic acid in 7,12-dimethylbenz(a)anthracene (DMBA) induced skin carcinogenesis in Swiss albino mice. Indian J. Exp. Biol..

[112] Venkatachalam K., Gunasekaran S., Jesudoss V.A.S., Namasivayam N. (2013). The effect of rosmarinic acid on 1,2-dimethylhydrazine induced colon carcinogenesis. Exp. Toxicol. Pathol..

[113] Baldasquin-Caceres B., Gomez-Garcia F.J., López-Jornet P., Castillo-Sanchez J., Vicente-Ortega V. (2014). Chemopreventive potential of phenolic compounds in oral carcinogenesis. Arch. Oral Biol..

[114] Furtado R.A., Oliveira B.R., Silva L.R., Cleto S.S., Munari C.C., Cunha W.R., Tavares D.C. (2015). Chemopreventive effects of rosmarinic acid on rat colon carcinogenesis. Eur. J. Cancer Prev. Off. J. Eur. Cancer Prev. Organ. ECP.

[115] Romo Vaquero M., García Villalba R., Larrosa M., Yáñez-Gascón M.J., Fromentin E., Flanagan J., Roller M., Tomás-Barberán F.A., Espín J.C., García-Conesa M.-T. (2013). Bioavailability of the major bioactive diterpenoids in a rosemary extract: Metabolic profile in the intestine, liver, plasma, and brain of Zucker rats. Mol. Nutr. Food Res..

[116] Doolaege E.H.A., Raes K., Vos F.D., Verhé R., Smet S.D. (2011). Absorption, Distribution and Elimination of Carnosic Acid, A Natural Antioxidant from *Rosmarinus officinalis*, in Rats. Plant Foods Hum. Nutr..

[117] Karthikkumar V., Sivagami G., Viswanathan P., Nalini N. (2015). Rosmarinic acid inhibits DMH-induced cell proliferation in experimental rats. J. Basic Clin. Physiol. Pharmacol..

[118] Romo-Vaquero M., Larrosa M., Yáñez-Gascón M.J., Issaly N., Flanagan J., Roller M., Tomás-Barberán F.A., Espín J.C., García-Conesa M.-T. (2014). A rosemary extract enriched in carnosic acid improves circulating adipocytokines and modulates key metabolic sensors in lean Zucker rats: Critical and contrasting differences in the obese genotype. Mol. Nutr. Food Res..

[119] European Food Safety Authority (EFSA) (2008). Use of rosemary extracts as a food additive—Scientific Opinion of the Panel on Food Additives, Flavourings, Processing Aids and Materials in Contact with Food. EFSA J..

[120] Pérez-Sánchez A., Barrajón-Catalán E., Caturla N., Castillo J., Benavente-García O., Alcaraz M., Micol V. (2014). Protective effects of citrus and rosemary extracts on UV-induced damage in skin cell model and human volunteers. J. Photochem. Photobiol. B.

[121] Romo-Vaquero M., Selma M.-V., Larrosa M., Obiol M., García-Villalba R., González-Barrio R., Issaly N., Flanagan J., Roller M., Tomás-Barberán F.A. (2014). A Rosemary Extract Rich in Carnosic Acid Selectively Modulates Caecum Microbiota and Inhibits β-Glucosidase Activity, Altering Fiber and Short Chain Fatty Acids Fecal Excretion in Lean and Obese Female Rats. PLoS ONE.

